# The composition of the perinatal intestinal microbiota in cattle

**DOI:** 10.1038/s41598-018-28733-y

**Published:** 2018-07-11

**Authors:** Mohammad Jaber Alipour, Jonna Jalanka, Tiina Pessa-Morikawa, Tuomo Kokkonen, Reetta Satokari, Ulla Hynönen, Antti Iivanainen, Mikael Niku

**Affiliations:** 1 Department of Veterinary Biosciences, Faculty of Veterinary Medicine, University of Helsinki, Helsinki, Finland; 2 Immunobiology Research Program, Faculty of Medicine, University of Helsinki, Helsinki, Finland; 3 Department of Agricultural Sciences, Faculty of Agriculture and Forestry, University of Helsinki, Helsinki, Finland

## Abstract

Recent research suggests that the microbial colonization of the mammalian intestine may begin before birth, but the observations are controversial due to challenges in the reliable sampling and analysis of low-abundance microbiota. We studied the perinatal microbiota of calves by sampling them immediately at birth and during the first postnatal week. The large size of the bovine newborns allows sampling directly from rectum using contamination-shielded swabs. Our 16S rDNA data, purged of potential contaminant sequences shared with negative controls, indicates the existence of a diverse low-abundance microbiota in the newborn rectal meconium and mucosa. The newborn rectal microbiota was composed of Firmicutes, Proteobacteria, Actinobacteria and Bacteroidetes. The microbial profile resembled dam oral rather than fecal or vaginal vestibular microbiota, but included typical intestinal taxa. During the first postnatal day, the rectum was invaded by *Escherichia/Shigella* and *Clostridia*, and the diversity collapsed. By 7 days, diversity was again increasing. In terms of relative abundance, Proteobacteria were replaced by Firmicutes, Bacteroidetes and Actinobacteria, including *Faecalibacterium*, *Bacteroides*, *Lactobacillus*, *Butyricicoccus* and *Bifidobacterium*. Our observations suggest that mammals are seeded before birth with a diverse microbiota, but the microbiota changes rapidly in the early postnatal life.

## Introduction

Microbial symbionts are essential for the development and physiology of mammals. In the gut, a diverse microbiota characteristic of each host species drives the proper differentiation and function of the immune system and mucosal membranes^1–3^. A stable commensal community protects the host from invasive pathogens^4^. The microbiota also expands the host’s biosynthetic capacity and enhances nutrient uptake^5^. This is most pronounced in ruminants, which rely on the ruminal microbiota to convert dietary fibers into compounds usable to mammalian cells^6^.

The first microbial inoculums are vertically transmitted from the mother to the offspring^7^. Major colonization starts at birth and is complemented during lactation^8^ and later life. Recent studies indicate that microbes play important roles already during the fetal development^9^. The offspring is affected by the pregnant mother’s microbial environment. Mother’s exposure to a diverse microbiota appears to be beneficial^10^, while antimicrobial treatments during pregnancy may increase the risk of immunological and metabolic disorders in the offspring^11^. Recently, microbe-derived compounds were elegantly shown to promote the differentiation of gut-specific innate lymphoid cells in the murine fetus^12^. However, it is still unclear whether intact bacteria or only their components or secreted products reach the fetus.

The mammalian fetus has generally been regarded as sterile until birth ever since the original research of the infant microbiota by Theodor Escherich^13^. However, bacteria were quite commonly detected in newborn human meconium already in subsequent early studies with culture and microscopy methods^14–17^. More recently, bacteria have been observed in newborn meconium using culturing and various DNA and RNA based methods including PCR, fluorescence *in situ* hybridization and high-throughput sequencing^18–21^. In addition to humans, microbes have been found in newborn calf meconium^22^, pregnant bovine uterus^23^ and foal amniotic fluid^24^. Enterococci administered orally to pregnant mice could be detected in fetal meconium^25^. These observations are still controversial due to technical challenges in reliable sampling and analysis of very low-abundance microbiota in these samples^26^. Human meconium samples are usually collected from passed meconium, often several hours after birth, and may be affected by early postnatal colonization. Direct culture, especially if coupled with bacterial enrichment, may allow sensitive detection of the low number of microbes, but optimizing the culture conditions requires prior knowledge of the microbes to be cultured. DNA-based analyses are highly sensitive in detecting a wide range of microbes without prior knowledge of their identity and growth conditions, but few of the published studies have reported proper controls, which are necessary to exclude the effects of microbial DNA present in molecular biology reagents and laboratory equipment.

In this study, we wanted to reliably address the *in utero* microbial colonization of mammals, using sampling and analysis methods optimizing contamination control. We sampled bovine rectal microbiota immediately at birth. The large size of the calves enabled sampling of meconium and mucosa directly from rectum using double sheathed sterile swabs, which were exposed only within the intestine. We performed 16S rDNA amplicon sequencing using high-quality DNA extraction reagents. Sampling device controls were included in the analysis, and microbial sequences shared with the controls were purged from the data, to minimize false positive observations. The development of the rectal microbiota was then followed to 7 days of age, and the microbial DNA profiles were also compared between calves and their dams.

## Results

### Quantification of bacterial DNA and contamination control

**Figure 1:**
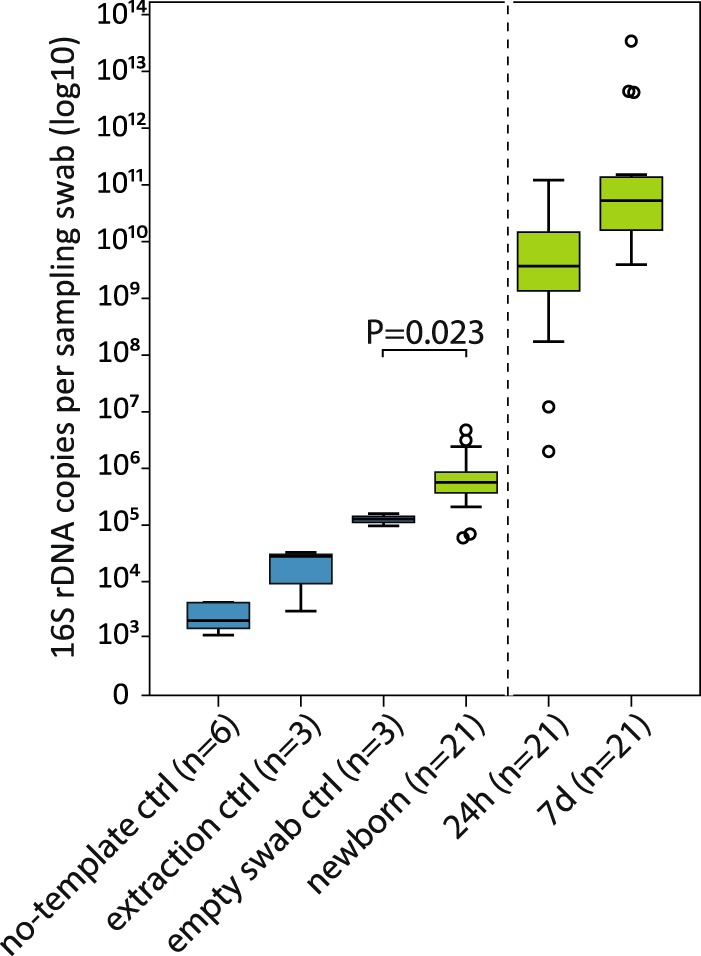
16S rDNA copy numbers per sample. Blue = negative controls, green = calf rectal sampling swabs. Values represent 16S rDNA copy numbers per sampling swab or the corresponding amount of extraction reagent (extraction control) or ultra pure H_2_O (no-template control). 24 h and 7 d samples were diluted more before amplification, in order to fit in the qPCR dynamic range (dashed line). The boxes represent the interquartile ranges (IQR) containing the middle 50% of cases. The horizontal line in a box indicates the median. Whiskers show maxima and minima within 1.5× IQR. Circles indicate outliers between 1.5–3× IQR.

The newborn samples (n = 21) were collected at birth directly from the rectum, using double-sheathed sampling swabs preventing external contamination. However, DNA extraction and PCR reagents always contain small amounts of bacterial DNA^27^. We quantified bacterial DNA in our samples and several levels of negative controls by qPCR using universal bacterial 16S rDNA primers and probe^28^. The total level of contaminating bacterial 16S rDNA was 1.3·10^5^ ± 3.1·10^4^ copies per swab (median ± SD, n = 3), which includes the contamination in the ultra-pure water, DNA extraction and PCR reagents and in the sterile swab itself. In the newborn rectal samples, there were 5.7·10^5^ ± 1.1·10^5^ copies of 16S rDNA per sampling swab (Fig. 1). Thus, the median copy number measured from newborn rectal swabs was 4.4 times the median copy number in the empty sterile control swabs (P = 0.023).

The bacterial load increased rapidly after birth (Fig. 1). Already at 24 hours, there were on average 7000 times more copies of the 16S rRNA gene compared to the newborns. By 7 days, there was a further 14-fold increase in comparison to the 24 h samples.

### Removal of potential contaminant sequences from 16S rDNA amplicon sequencing data

The microbiota composition of all collected samples as well as various negative controls was analyzed by amplifying the hypervariable regions of the 16S rRNA genes in the samples, followed by MiSeq sequencing. For conciseness, we will use the term “microbiota” throughout the text, although 16S rDNA amplicon sequencing does not directly prove the existence of live bacteria. Due to the sampling technique utilizing rectal swabs, the calf samples represented a mixture of luminal (meconial or fecal) microbiota and mucosal microbiota.

To minimize the likelihood of false positive observations, we removed from the sequencing data all microbial genotypes (16S rDNA sequences) which were abundant in the negative controls. The decontamination was performed using a previously published logic as described in Methods and Supplementary information^29^. The sample and control data were compared at the level of de-noised 16S rDNA sequences, in order to maximally utilize the resolution of the original data and to distinguish related but different species in samples and controls.

**Figure 2:**
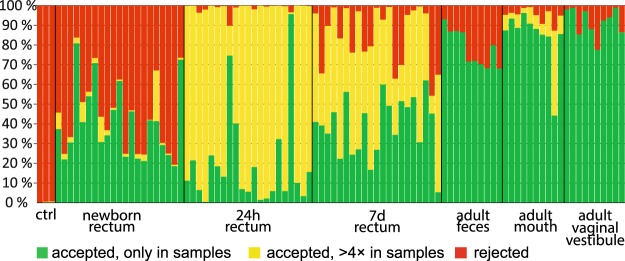
Effect of data decontamination on the animal microbiota samples. Each bar represents all sequence reads obtained from one sample. Green color shows the proportion of genotypes (de-noised 16S rDNA sequences) found only in the actual samples. Yellow shows genotypes which were shared between samples and negative controls, but were accepted due to more than four-fold relative abundance in samples. Red shows sequences which were removed due to their abundance in the controls.

In the newborn samples, 41 ± 18% of sequence reads (average ± SD) represented genotypes that were not detected in any of the negative controls (green bars in Fig. 2). In addition, 4 ± 6% of the newborn reads were accepted although identical sequences were present in negative controls, as their relative abundance was more than four times higher in the actual samples. 55 ± 19% of newborn reads represented genotypes abundant in the negative controls and were thus discarded as potential contaminants. Thus, a larger proportion of the newborn data was deleted than what was indicated by the quantification of contamination levels by qPCR, suggesting that some sequences actually originating from the samples were lost (Supplementary Fig. 1).

Finally, all rare OTUs (detected less than 250 times in the entire decontaminated dataset) were excluded. These represented 3.2% of the sequence reads in the entire dataset (on average, 6.9 ± 8.4% of sequences in individual samples).

In the 24 h and 7 d samples, a large proportion of the reads represented genotypes that were shared with the negative controls, but at more than six times smaller relative abundance (shown as yellow in Fig. 2). This is due to the low diversity of these samples. A single genotype of the *Escherichia/Shigella* genus generated 68% of all of the reads in the 24 h samples in average and was also found to be very frequent in the 7 d samples. However, as the total number of 16S rDNA copies in the 24 h samples was, on average, >100000 times higher than in the negative controls, it is highly unlikely that this genotype was a reagent/sampling device contaminant.

The final quality-controlled, decontaminated and rarity-filtered dataset contained in total 4078141 sequences. The original 16S rDNA sequencing data is available in the NCBI SRA database with the accession number SRP128833. The median sequence read, OTU and genera counts per sample type are shown in Table 1. All detected taxa are presented in Supplementary Dataset 1.

**Table 1:** Characteristics of the 16S rDNA amplicon sequencing data.

	Newborn rectum	24 h rectum	7 d rectum	Dam feces	Dam mouth	Dam vaginal vestibule	Empty swab ctrl
n	21	21	21	10	10	10	3
Reads, raw	21342 (11198)	76993 (39128)	47755 (16912)	25579 (3453)	51539 (15477)	39464 (13936)	4486 (3449)
Reads, decont.	7127 (10659)	76854 (39164)	36008 (9917)	24617 (3633)	48907 (15670)	38894 (14024)	44 (30)
OTU, raw	142 (66)	68 (78)	72 (30)	335 (17)	339 (118)	362 (35)	78 (17)
OTU, decont.	100 (66)	40 (63)	54 (12)	322 (12)	275 (120)	336 (32)	4 (4)
Genera, raw	94 (6)	37 (16)	39 (26)	74 (12)	136 (31)	95 (19)	58 (9)
Genera, decont.	64 (5)	22 (17)	26 (22)	58.5 (5)	112 (28)	70.5 (12)	4 (3.5)
Actinobacteria %	11 (16)	0 (0.18)	2.8 (6.2)	0.40 (0.16)	3.1 (3.0)	1.0 (3.2)	
Bacteroidetes %	5.2 (13)	0 (0.49)	12 (17)	20 (5.2)	2.4 (4.4)	7.7 (8.8)	
Firmicutes %	39 (22)	22 (20)	80 (28)	76 (5.1)	62 (9.7)	83 (10)	
Proteobacteria %	30 (18)	78 (20)	0.38 (15)	0.42 (0.41)	33 (12)	0.70 (1.9)	

Medians and standard deviations (in parentheses) for sequence reads, OTU and genus level taxa numbers in raw and decontaminated data, and relative abundances of major phyla in decontaminated data are shown.

### Characterization of the newborn rectal microbiota

**Figure 3:**
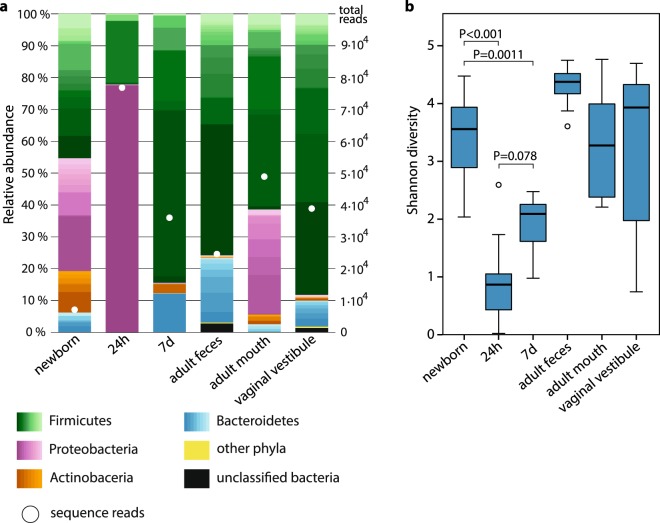
Microbiota composition and microbial diversity in calves (n = 21) and adults (n = 10). (**a**) Median microbiota compositions (scaled to totals of 100%) and sequence numbers. Within phyla, the relative abundances of individual genera are shown with different shades. The lightest shades in each phylum show the combined abundance of the least abundant genera (<0.5% of total). For sequencing, control and newborn samples were preamplified by 21 PCR cycles, 7-day and adult feces by 15, and others by 18 to compensate for different quantities of 16S rDNA copies in the samples. (**b**) Shannon diversity. Boxplot as in Fig. 1.

The newborn rectal microbiota was dominated by Firmicutes, Proteobacteria, Actinobacteria and Bacteroidetes (Fig. 3a and Table 1). Despite the low bacterial load in newborns, the alpha diversity was higher than in the older calves (P < 0.005; Fig. 3b and Supplementary Fig. 2). This was also evident when examining the dominant phyla separately at genus level, where the diversity within the dominant phyla was highest in newborns (Supplementary Figs 3–6).

Genus-level core taxa (Table 2) were defined as being present in at least 75% of newborns and at >0.1% median abundance; these represented a median of 36% of all individual genus-level taxa and 64% of all sequence reads per newborn rectal sample.

**Table 2:** Core bacterial taxa (>0.1% relative abundance, >75% prevalence) in newborn rectal swabs.

Taxon	Abundance	SD	Prevalence
Firmicutes			
*Bacilli; Bacillales; Staphylococcaceae; Staphylococcus*	2.5%	9.3%	100%
*Bacilli; Lactobacillales; Lactobacillaceae; Lactobacillus*	0.7%	3.3%	95%
*Bacilli; Lactobacillales; Streptococcaceae; Streptococcus*	2.7%	7.1%	95%
*Clostridia; Clostridiales; Lachnospiraceae; Clostridium-XlVa*	0.1%	0.3%	76%
*Clostridia; Clostridiales; Peptostreptococcaceae; Clostridium-XI*	0.6%	1.9%	76%
*Clostridia; Clostridiales; Peptostreptococcaceae; Romboutsia*	0.7%	2.6%	76%
*Clostridia; Clostridiales;* unclassified *Lachnospiraceae*	1.1%	1.8%	95%
*Clostridia; Clostridiales;* unclassified *Ruminococcaceae*	2.2%	2.6%	95%
*Clostridia;* unclassified *Clostridiales*	0.6%	0.8%	76%
Proteobacteria			
*Alphaproteobacteria; Caulobacterales; Caulobacteraceae; Brevundimonas*	0.2%	0.5%	86%
*Alphaproteobacteria; Rhodobacterales; Rhodobacteraceae; Paracoccus*	0.6%	0.6%	86%
*Alphaproteobacteria; Sphingomonadales; Sphingomonadaceae; Sphingomonas*	0.6%	0.5%	100%
*Betaproteobacteria; Burkholderiales; Comamonadaceae; Comamonas*	0.2%	0.5%	76%
*Betaproteobacteria; Burkholderiales; Oxalobacteraceae; Massilia*	0.7%	11.7%	90%
*Betaproteobacteria; Neisseriales;* unclassified *Neisseriaceae*	0.6%	1.3%	90%
*Gammaproteobacteria; Enterobacteriales;* unclassified *Enterobacteriaceae*	0.9%	4.5%	95%
*Gammaproteobacteria; Pasteurellales;* unclassified *Pasteurellaceae*	0.1%	3.1%	81%
*Gammaproteobacteria; Pseudomonadales; Moraxellaceae; Acinetobacter*	6.6%	5.2%	100%
*Gammaproteobacteria; Pseudomonadales; Pseudomonadaceae; Pseudomonas*	2.7%	6.1%	95%
Actinobacteria			
*Actinobacteria; Actinomycetales; Corynebacteriaceae; Corynebacterium*	2.8%	14.9%	100%
*Actinobacteria; Actinomycetales; Dermabacteraceae; Brachybacterium*	0.5%	0.7%	95%
*Actinobacteria; Actinomycetales; Micrococcaceae; Kocuria*	1.0%	2.5%	95%
*Actinobacteria; Actinomycetales; Micrococcaceae; Micrococcus*	0.7%	3.3%	86%
*Actinobacteria; Actinomycetales; Micrococcaceae; Rothia*	0.1%	0.5%	86%
Bacteroidetes			
*Bacteroidia; Bacteroidales; Bacteroidaceae; Bacteroides*	0.6%	2.0%	90%
Unclassified Bacteroidetes	0.4%	1.4%	76%

Median relative abundances, standard deviations and prevalence in 21 calves are shown.

**Figure 4:**
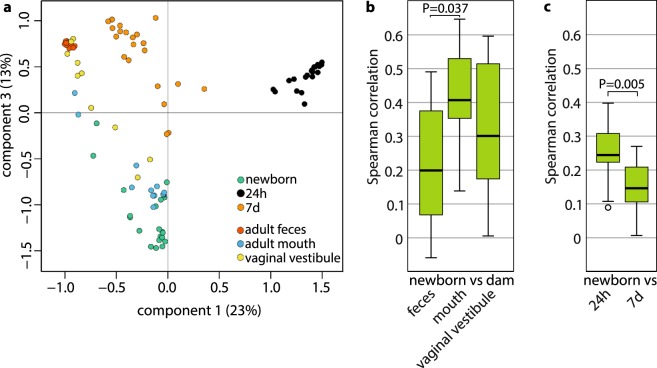
(**a**) Principal coordinates analysis (PCoA) of newborn rectal microbiota and dam fecal, oral and vaginal vestibular microbiota. The sample type explained 53% of the variance across all samples. n = 21 calves and 10 cows. (**b**) Similarity of microbiota in newborns and their own dams, expressed as Spearman correlations (ρ). Boxplot as in Fig. 3; n = 10 calf-dam pairs. (**c**) Similarity of microbiota in newborns and the same calves at 24 h and 7 d (ρ). n = 21 calves.

We evaluated the similarity of the microbiota profiles in newborns, older calves and various adult cow locations by principal coordinates analysis (PCoA; Fig. 4a) and Spearman rank correlations (ρ; Fig. 4b,c). In PCoA, the newborn rectal microbiota clustered closest to adult oral microbiota (Fig. 4a). The correlations between newborns and the oral, fecal and vaginal microbiotas of their own dams (Fig. 4b) were significantly different (Friedman χ^2^ = 6.2; df = 2; P = 0.045). The correlation was strongest between newborns and the oral microbiota of their own dams (median ρ = 0.41; SD = 0.16; n = 10). This was significantly higher (P = 0.037) than the correlation between newborns and dam fecal microbiota (median ρ = 0.20; SD = 0.17; n = 10). The correlation between newborns and dam vaginal vestibular microbiota (median ρ = 0.30; SD = 0.20; n = 10) did not differ significantly from these. The newborn rectal microbiota was significantly more similar to the 24 h rectal microbiota than to the 7 d rectal microbiota (P = 0.005; Fig. 4c). The newborns were not significantly more similar to their own dams, compared to the other cows (not shown).

**Figure 5:**
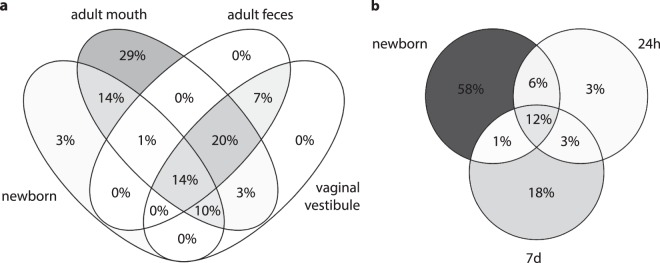
Shared genus-level bacterial taxa. (**a**) Taxa shared between newborns and various dam samples (total: 136 taxa). (**b**) Taxa shared between newborns and older calves (total: 73 taxa). For each sample group, all taxa with a median abundance >0 were included. Note that a larger number of taxa were shared at lower abundance levels (see text).

This similarity between the newborn and dam oral microbiota was also evident when comparing the genus level microbial composition (Supplementary Figs 3–6). As a group, newborns shared more genus-level taxa with adult oral microbiota (39% of taxa with positive median abundance in any sample group) than with adult vaginal vestibular microbiota (24%) or fecal microbiota (15%) (Fig. 5a). Notably, 14% of the taxa were exclusively shared between newborn and adult oral microbiota, but none were exclusively shared between newborns and adult vaginal vestibule or adult feces.

**Figure 6:**
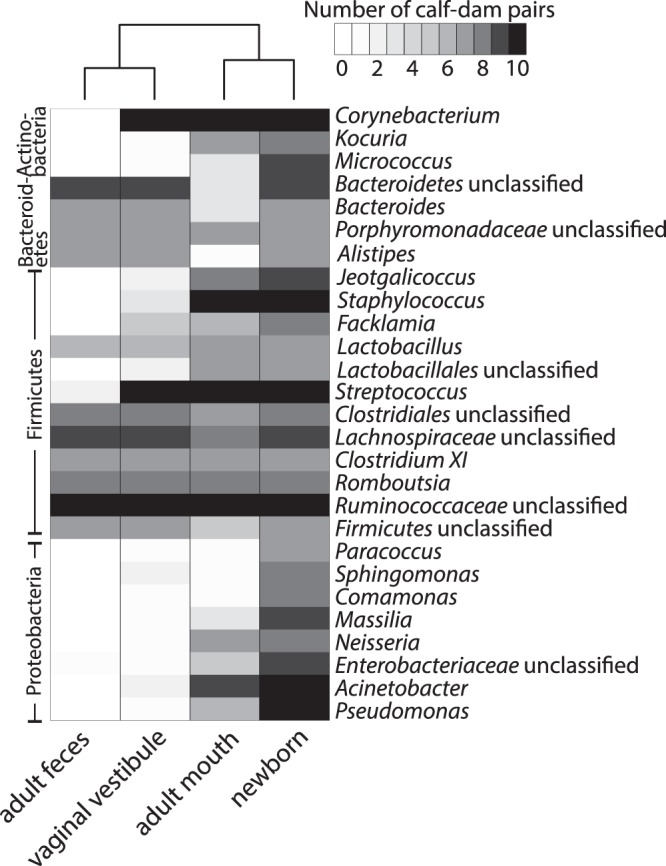
Co-existence of genus-level bacterial taxa in newborns and their own dams. The heatmap shows the occurrence of each taxon in the feces, vaginal vestibule and mouth of the cow, if detected in the cow’s own calf (cutoff: ≥20 hits/taxon/animal), and the occurrence of each taxon in the calves. Only the taxa present in >50% of the newborns are shown.

We also studied the co-existence of genus-level bacterial taxa in the newborns and their own dams. All the newborn rectal core genera of the Firmicutes and Bacteroidetes phyla were found in at least one location of the dam, if present in its calf. Bacterial taxa co-existed in the newborn and the mouth of its own dam more frequently than in the newborn and the vaginal vestibule or feces of the dam (Fig. 6). This was most evident for Proteobacteria and Actinobacteria.

### Early postnatal development of the fecal microbiota

Already 24 hours after birth, the rectal microbiota composition was dramatically different in comparison to the newborns (Fig. 4a). The low diversity microbiota (Fig. 3a,b and Supplementary Fig. 2) contained only Proteobacteria and Firmicutes (Table 1), although the bacterial load was significantly higher (Fig. 1). In the 24 h samples, 77% of all sequences were of the *Escherichia/Shigella* genus (Table 3). Uncultured *Clostridium* taxa (sensu stricto) and *Enterococcus* were also found in all of these samples, with median relative abundances of 8% and 0.8%, respectively.

**Table 3:** Core bacterial taxa (>0.1% relative abundance, >75% prevalence) in 24 h calf rectum.

Taxon	Abundance	SD	Prevalence
Proteobacteria			
*Gammaproteobacteria; Enterobacteriales; Enterobacteriaceae; Escherichia/Shigella*	76.8%	24.87%	100%
*Gammaproteobacteria; Enterobacteriales;* unclassified *Enterobacteriaceae*	0.4%	8.21%	90%
Firmicutes			
*Bacilli; Lactobacillales; Enterococcaceae; Enterococcus*	0.8%	7.51%	100%
*Bacilli; Lactobacillales; Streptococcaceae; Streptococcus*	0.2%	4.11%	95%
*Clostridia; Clostridiales; Clostridiaceae-1; Clostridium-sensu-stricto*	8.2%	14.38%	100%

Median relative abundances, standard deviations and prevalence in 21 calves are shown.

Of the newborn rectal core taxa, only *Streptococcus* and unclassified *Enterobacteriaceae* were abundant one day after birth. All other core Proteobacteria and all core Actinobacteria were undetectable at 24 h in most calves. However, we detected most newborn core Firmicutes and *Bacteroides* at this time point in >50% of the calves which were positive at birth (Supplementary Table 3).

Of all the genus-level taxa with a positive median abundance in newborns or 24 h old calves, 23% were shared between these age groups (Fig. 5b). Four new taxa with a positive median abundance were observed after birth, during the first postnatal day. In addition to *Escherichia/Shigella*, these included *Terrisporobacter*, some unclassified *Clostridiaceae* and *Butyricicoccus*.

Newborn and 24 h samples from the same calves were not more similar than newborn and 24 h samples from different calves (not shown). The 24 h samples were also not significantly more similar to their own dams than to the other cows. By Spearman correlations, the calf rectal microbiota at 24 h was still slightly more similar to dam oral microbiota (median ρ = 0.27; SD = 0.15; n = 10) than to dam fecal microbiota (median ρ = 0.23; SD = 0.13; n = 10) or dam vaginal vestibular microbiota (median ρ = 0.18; SD = 0.10; n = 10). These differences were statistically significant (Friedman χ^2^ = 7.8; df = 2; P = 0.02). Again, the correlation between calf rectal and dam oral microbiota was significantly higher than between calf rectal and dam fecal microbiota (P = 0.02), but the correlation between calf rectal and dam vaginal vestibular microbiota did not differ significantly from these.

At 7 days after birth, the rectal microbiota composition started to resemble that of the adult feces. The correlation between 7-day calf rectal and dam fecal or vaginal microbiota (for both comparisons, median ρ = 0.28; SD = 0.06; n = 10) was significantly higher than the correlation between calf rectal and dam oral microbiota (median ρ = 0.15; SD = 0.09; n = 10; Friedman χ^2^ = 9.8; df = 2; P = 0.007; pairwise P < 0.05). However, the 7-day calf rectal microbiota contained more Actinobacteria than adult feces (P = 0.04; Fig. 3a and Table 1). The raw data from 7-day rectal microbiota also contained more Proteobacteria, mostly of the *Escherichia/Shigella* genus (Supplementary Fig. 1). These were removed in the data decontamination, but probably actually exist in the 7-day calf rectum, as the decontamination protocol did not take into account the much higher 16S rDNA copy numbers in these samples as compared to the negative controls. By 7 days, alpha diversity had increased from the 24 h samples in 18 out of 21 calves, but was in all cases still clearly below the newborn samples (Fig. 3b and Supplementary Fig. 2). The most abundant genera, found in all calves 7 d after birth, were *Faecalibacterium, Bacteroides, Lactobacillus* and *Butyricicoccus* (Table 4).

**Table 4:** Core bacterial taxa (>0.1% relative abundance, >75% prevalence) in 7 d calf rectum.

Taxon	Abundance	SD	Prevalence
Firmicutes			
*Bacilli; Lactobacillales; Lactobacillaceae; Lactobacillus*	8.3%	8.21%	100%
*Bacilli; Lactobacillales; Streptococcaceae; Streptococcus*	0.1%	24.87%	95%
*Clostridia; Clostridiales; Lachnospiraceae; Clostridium-XlVa*	2.0%	8.21%	90%
*Clostridia; Clostridiales; Ruminococcaceae; Butyricicoccus*	3.8%	24.87%	100%
*Clostridia; Clostridiales; Ruminococcaceae; Faecalibacterium*	27.5%	24.87%	100%
*Clostridia; Clostridiales;* unclassified *Lachnospiraceae*	1.5%	24.87%	90%
*Clostridia; Clostridiales;* unclassified *Ruminococcaceae*	1.0%	8.21%	95%
Bacteroidetes			
*Bacteroidia; Bacteroidales; Bacteroidaceae; Bacteroides*	11.6%	17.37%	100%
*Bacteroidia; Bacteroidales; Porphyromonadaceae; Parabacteroides*	0.1%	0.51%	81%
Actinobacteria			
*Actinobacteria; Bifidobacteriales; Bifidobacteriaceae; Bifidobacterium*	2.6%	6.13%	95%

Median relative abundances, standard deviations and prevalence in 21 calves are shown.

Of the newborn core taxa, *Streptococcus*, unclassified *Ruminococcaceae*, unclassified *Lachnospiraceae* and *Clostridium* XIVa were also abundant at 7 days. In addition, most other Firmicutes and *Bacteroides* were detected at some level in >50% of calves which were positive at birth (Supplementary Table 3). Newborn core Actinobacteria and Proteobacteria were only detectable in less than half of the calves.

More than half of the genus-level taxa with positive median abundances in 7 d samples reached this level only after the first 24 hours (Fig. 5b). In addition to *Faecalibacterium*, these included *Bifidobacterium*, *Parabacteroides* and *Collinsella*.

A detailed characterization of the adult fecal, oral and vaginal vestibular microbiota is available in the Supplementary information (Supplementary Tables 4–6 and Supplementary Figs 3–6).

## Discussion

It is still unclear whether host-microbe interactions in mammals are initiated after birth or already during fetal development^30^. This is the first published study characterizing the newborn bovine intestinal microbiota with sequencing based methods. The bovine calves allowed an especially reliable investigation of the initial mammalian microbiota, as their large size enabled sampling immediately at birth and directly within rectum, using contamination-protected swabs. We also rigorously filtered the sequencing data, removing sequences which were shared between samples and negative controls and thus potentially represented contaminant microbial DNA originating from reagents and instruments.

Our results suggest that the late fetal intestine already contains a low-abundance but diverse microbiota, obtained *in utero* from the mouth or proximal digestive tract of the dam. This is in line with recent research on human newborn and placental microbiota^18,31,32^. Within 24 hours from birth, the abundance of rectal microbes increased dramatically, but alpha diversity collapsed. At the age of one week, the overall composition of the rectal microbiota was already closer to the adult feces, but still much lower in diversity.

The newborn rectal microbial 16S rDNA profile was dominated by the presence of Firmicutes and Proteobacteria, with smaller contributions of Actinobacteria and Bacteroidetes. The observed core microbiota (>0.1% relative abundance, >75% prevalence) represents a mixture of taxa containing known anaerobic gut microbes (*Ruminococcaceae, Clostridium* clusters XIVa and XI, unclassified *Lachnospiraceae, Romboutsia, Bacteroides*), unclassified *Enterobacteriaceae* and typical initial gut colonizers or bacteria found in the intestine but more typically present on other mucosae (*Streptococcus, Staphylococcus, Corynebacterium, Kocuria, Micrococcus*). Several of these genera have been previously observed in human meconium^18,31,32^. *Acinetobacter*, the most abundant single genus in our newborn data, has been previously detected in bovine small intestinal mucosa^33^ and is hypothesized to contribute to the regulation of epithelial renewal in the crypts^34^. *Sphingomonas*, another aerobic genus detected in every newborn, produces glycosphingolipids modulating the phenotype and function of intestinal invariant natural killer T (iNKT) cells in mice^35^.

In the newborn samples, several taxa from the *Lactobacillales* order were abundant. This replicates previous findings where Lactococci and *Leuconostoc* were detected in calf meconium collected immediately after birth^22^. The pregnant cow endometrial microbiota was recently characterized by 16S rDNA amplicon sequencing^23^, and the most abundant bacterial families (*Porphyromonadaceae*, *Ruminococcaceae, Lachnospiraceae* and *Bacteroidaceae*) found in that study were also detected in most of our newborn calves. In a recent study by Costa *et al*.^36^, the newborn foal meconial microbiota resembled our observations: being mostly composed of Firmicutes and Proteobacteria, it included *Clostridiales*, *Ruminococcaceae*, *Lachnospiraceae* and *Lactobacillus*. The authors did not specify the exact timing of sample collection.

Bovine newborns possess several favorable characteristics for reliable investigations of the early mammalian intestinal microbiota. The gestation period is very similar to humans, thus allowing more time for the development of a fetal microbiota and associated immune responses than possible in mice. The size of the calves allows the use of contamination-protected sampling swabs, which are too large for human newborns. Research in humans relies on passed meconium samples, collected several hours from birth, and are therefore more comparable to our 24 h samples, discussed below. We performed the sampling within minutes from birth, making it unlikely that the microbiota within rectum would have been significantly affected by external bacteria. In contrast to humans and mice, bovine calves are enclosed in double fetal membranes (chorioallantoic and amniotic membranes) and are thus isolated from the environment until the final stages of labor. When the inner membrane ruptures, the anal end of the calf is still located deep in the clean uterus, one meter from the external orifice. The final expulsion of the calf from this position is rapid and occurs usually within 30 min to 2 hours from the rupture of the membranes. In the conventionalization of germ-free mice, very few bacterial species are detected within the first hours of exposure^37^. In our data, the comparison of the newborn rectal microbiota with the vaginal vestibular microbiota does not suggest that the microbial DNA observed in the newborns originates from the reproductive tract. None of the newborn rectal core genera of the Proteobacteria group and only one genus of the rectal core Actinobacteria were detected in the vestibule in >50% of cows.

Despite the advantages of using cattle, the analysis of low-abundance microbiotas is challenging due to microbial DNA contamination present in equipment and reagents. We used sterile disposable microbiological sampling devices and high-quality DNA extraction and PCR reagents, and performed a rigorous de-contamination of the 16S rDNA sequence data, based on sequencing of empty sampling device controls. While it is not possible to exclude all potential contaminants with absolute certainty, the most prevalent and abundant bacterial genera observed in newborns were mostly known animal mucosal commensals as expected. Some of the detected taxa are common reagent and laboratory contaminants^27^, which as such does not exclude them from being of animal origin in the samples. Some of the observed core genera (such as *Massilia*) have been isolated from environmental sources and could thus represent either persistent contamination, previously uncharacterized animal-associated species or transient colonizers of the dam originating from its feed.

The stringent decontamination protocol probably excluded some taxa which actually existed in the animals. This is suggested by the higher proportion of rejected sequences as compared to the qPCR-measured ratio of 16S rDNA copy numbers in negative controls and samples. Taxa shared between samples and controls were deleted if they were relatively abundant in the controls. Due to the small total sequence read counts in the negative controls, a small absolute number of reads could result in a high relative abundance. Furthermore, although we utilized the full resolution of 16S rDNA sequencing, it is not always sufficient to distinguish strains or even closely related species in samples and controls. Such false negatives are an unavoidable consequence of maximizing the reliability of the bacteria we report in the newborn gut, and mean that we probably underestimated the diversity of the newborn microbiota.

Sources and translocation mechanisms of fetal microbiota are currently not understood. Our comparison of newborn and dam samples suggests that the newborn rectal microbiota originates from the mouth or proximal digestive tract of the dam. The similarity to dam oral microbiota was especially clear for Proteobacteria and Actinobacteria, but almost all newborn core taxa were detected in the mouth of >75% of cows. *Lactobacilli*, which were detected in majority of newborns, were infrequent in the bovine vagina, as observed previously^38^, but were the 2^nd^ most abundant genus in the cow oral microbiota in our data. In humans, similarities between the oral and placental microbiota have been also suggested^31,39^. Oral microbes are able to cross the mucosal barrier due to mechanical damage. Periodontal pathogen antigens have been detected in preterm placental tissues, and periodontal disease may be associated with adverse pregnancy outcomes^40^. In mice, bacteria administered orally to pregnant dams^25^, or metabolic products of those bacteria^12^, have been detected in the offspring.

We did not have the opportunity to sample dam forestomach or small intestine in this study, and therefore it is possible that the microbiotas in these regions of the digestive tract would have been even closer to the newborn rectal microbiota. The bovine oral microbiota reflects the contents of rumen, the major container of essential microbes in ruminants^41^. In a phylum level comparison to a previously published data on bovine digestive tract microbiota^33^, the newborn rectal microbial DNA profile in our calves resembles most that of the bovine small intestinal mucosa. In the previous study, *Acinetobacter* was the most abundant genus detected in the small intestinal mucosa, and *Ruminococcaceae*, *Lachnospiraceae*, *Enterobacteriaceae* and *Pseudomonas* were also abundant in the bovine small intestinal mucosa or digesta.

As the dam was only allowed to lick the calf after the first sampling, the oral bacteria observed in the newborn rectum must have arrived *in utero*. Active mechanisms are probably necessary to allow the translocation of microbes or microbial macromolecules from the dam to the fetus. Dendritic cells are able to collect live microbes from the gut and transport them at least to the mesenteric lymph nodes^42^, and maternal leukocytes have been shown to invade fetal tissues in mice^43^. Recently, bovine uterine pathogens were suggested to spread hematogenously^44^. The most obvious route from maternal blood to the fetus is through the placenta. Several recent studies have reported the existence of a low-abundance microbiota in healthy human placentae, also in samples obtained from sterile Caesarean sections^18,45^. The human placental microbiota was reported to include several bacterial genera detected in our newborn calves, but the overall composition may differ depending on species and even placental location analyzed.

The early microbes may serve to facilitate the establishment of a functional intestinal microbiota by several possible mechanisms. The first colonizers are thought to render the intestinal environment suitable to actual anaerobic intestinal microbes^46^. They also initiate the microbe-microbe and host-microbe interactions influencing the composition of the gut microbiota. Recent research suggests that a basic repertoire of immunoglobulin A (IgA) -producing B cells may be established very early in life, and will then regulate and stabilize the composition of the microbial community^47,48^. The initial IgA repertoire may be shaped in the fetus by the maternal microbiota. Maternal microbes could also induce tolerance for gut commensals. Tolerogenic regulatory T cells (T_regs_) are induced by commensal microbial antigens and microbial metabolites in the adult intestine^49,50^. This has not yet been investigated in the fetus, but maternal alloantigens have been shown to induce fetal T_regs_^43^.

Another potential function for prenatal host-microbe interactions is to stimulate the development of an effectively protective immune system. This may be especially important in ruminants. Due to the relative impermeability of the bovine epitheliochorial placenta, which prevents fetal immunoglobulin transfer, the newborn calf is completely dependent on its own premature immune system and the immunoglobulins obtained from the colostrum. The bovine germline immunoglobulin gene repertoire is very limited in size and diversity as compared to human and mouse, restricting the capacity to generate diverse antibodies by recombination^51,52^. We have previously shown that cattle compensate this by initiating immunoglobulin somatic hypermutation already during the fetal development^53^. This occurs in lymphoid follicles of the ileal Peyer’s patch, in the wall of the distal small intestine. Hypermutation catalyzed by activation-induced cytidine deaminase is generally presumed to be antigen-driven, and bioinformatic analysis of fetal bovine immunoglobulin sequences revealed signatures of selection^53^. Microbes provided by the mother may serve to initiate the early hypermutation.

We observed a massive increase in the bacterial load during the first day of postnatal life, and the relative composition of the microbial community changed dramatically. By 24 hours, the calf rectum was invaded by members of the facultatively anaerobic *Escherichia/Shigella* genus. The extreme *Escherichia/Shigella* dominance in young calves has been previously described^22^. In humans, this appears to be less pronounced, although the genus has also typically been observed in first-day human meconium^18,25^. Uncharacterized members of the *Clostridium* group (sensu stricto) were also very abundant in our calves already at 24 hours. In previous studies, clostridial species have appeared slightly later in calf fecal microbiota^22,54^. The facultative anaerobe *Enterococcus*, also typical in human meconium, was detected in all of our calves at 24 hours. *Clostridium* and *Enterococcus* were detected in most of our animals already at birth, but at <0.1% median abundance. Despite these changes, the correlation between the calf rectal microbiota and the oral microbiota of its own dam was still relatively high at 24 hours, as compared to dam fecal microbiota. The dams were allowed to lick their calves after the first sampling at birth, thus allowing transfer of oral microbes.

During the first week of life, the phylum-level composition of the rectal microbiota already changed towards the adult fecal microbiota, almost completely composed of Firmicutes and Bacteroidetes, indicating that the colonic environment had become anaerobic. In the relative composition data, Proteobacteria were largely replaced by anaerobic members of *Clostridiales* and *Bacteroidales* . At the genus level, the 7-day fecal microbiota was very different from adult feces, and the alpha diversity was still low. *Bifidobacterium* and especially *Lactobacillus* were abundant in the 7-day calf fecal microbiota, as expected for milk-consuming calves. However, they were much less abundant than what has been reported in breast-fed human infants, possibly reflecting the smaller concentrations and different composition of prebiotic oligosaccharides in the bovine milk^55^. In the 7-day calves, *Faecalibacterium* was the most abundant single genus, compatible with previous observations^56^. *F. prausnitzii* is an important commensal in humans, and also involved in the maintenance of intestinal epithelial homeostasis and mucus production in mice^57^. In our data, this genus was accompanied by another butyrate producer *Butyricicoccus*. Both of these were rare in the adult feces, as observed previously for forage-fed cattle, in contrast to those on corn-based diet^58^. Farming practices are known to impact the calf microbiota development. The calves in this study were separated from their dam at 24 hours and were individually housed for the first week, consuming mixed milk from the farm.

It is important to note that 16S rDNA amplicon sequencing does not allow actual tracking of the fates of specific microbial strains from newborns to later life. High-throughput sequencing is relative, and therefore early bacterial taxa may be overrun in the data by the multitude of postnatal colonizers, even if they actually persisted at low abundance. Furthermore, reliably distinguishing between persistence of an early colonizer and later colonization events by closely related microbes requires deep metagenomic sequencing and detailed analysis of strain-specific genetic variation^59^.

Taken together, our work adds to the accumulating evidence for prenatal host-microbe interactions occurring in healthy pregnancies and potentially instructing the developing immune system. An important remaining question is whether the fetal gut actually contains live, proliferating microbes, as PCR-based analyses may detect also fragmented extracellular DNA. The low microbial abundance in newborns, observed here and in previous studies, suggests that either microbial proliferation is strictly controlled in the fetus or only microbial components and metabolites reach it. Extremely high concentrations of bacteriostatic proteins have been reported in the human meconium^60^, suggesting a potential control mechanism, as well as potentially explaining some of the previously unsuccessful attempts in culturing meconium microbes. The information about the composition of the potential fetal microbiota will ease the selection of optimal growth media and conditions for future cultivation experiments. The data presented here can also aid in the design of targeted experiments aiming to define microbial components and metabolic products essential for the early immune development. Activation of the immune system and the establishment of tolerance could also be induced by microbial fragments, and therefore prenatal interactions with the maternal microbiota may be essential even if live microbes do not reach the fetus.

## Methods

### Animal fostering and sampling

The microbiological sampling was carried out at Viikki Research Farm, University of Helsinki. The study was reviewed and approved by the University of Helsinki Viikki Campus Research Ethics Committee and carried out in accordance with animal welfare guidelines and regulations.

**Figure 7:**
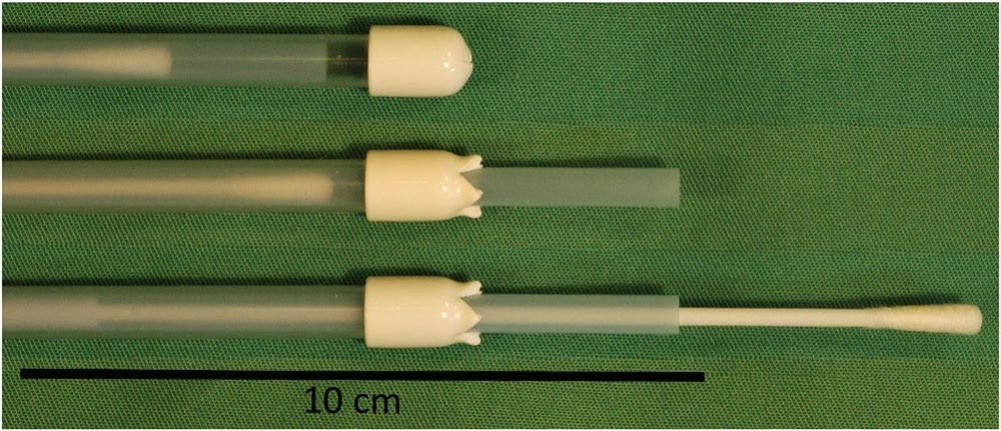
Rectal sampling swab. The sterile equine uterine sampling swab is protected by a double sheath, which prevents contamination at the anus. The sampling head was only exposed within the rectum and then in the laminar flow cabinet upon DNA extraction.

Rectal microbiota samples were collected from twenty-one Ayrshire and Holstein dairy cattle calves. The samples were collected using sterile double sheathed equine uterine culture swabs (EquiVet, Kruuse, Denmark; Fig. 7) at three time points: within 10 minutes from birth (newborn), 24 hours and 7 days after birth. The swab was only exposed within rectum and then in the laminar hood upon DNA extraction. All calves were born vaginally at full term. The dams were allowed to lick their calves after sampling. The calves were kept with their mothers for approximately 24 hours in the calving boxes. They were allowed to suck their dams or fed with 2 liters of colostrum within 4–6 hours from birth. After 24 hours, the calves were moved to individual calf pens and fed by pooled cow milk for the rest of the study period.

In addition, 10 of the cows were sampled for fecal, vaginal vestibular and oral microbiota shortly (0–12 days) before giving birth. The buccal and vestibular samples were collected by sterile cotton swabs (Applimed SA, Chatel-St-Denis, Switzerland) and immediately placed in sterile cryovials. The vestibular microbiota was sampled by opening the labia and sampling a single site as deep as visibly possible, close to the urethral opening. Dam fecal samples were taken rectally by using sterile surgical gloves over cattle rectal examination gloves. The cows were housed in tie-stalls on saw dust bedding until moving to calving boxes. A two-month dry period (without lactation) preceded the calving. During the early dry period cows were fed with grass silage. Grass silage was supplemented with 1 kg/d commercial concentrate (mainly barley, wheat and rape seed meal) for the last two weeks of pregnancy. In accordance with the farm’s mastitis prevention protocol, each quarter of the cow’s udder was treated once by 5 ml of intramammary suspension Umpimycin VET (Boehringer Ingelheim, Denmark) 7–8 weeks before calving. Each dose contained 100 mg penethamate hydroiodide, 280 mg benethamine penicillin and 100 mg framycetin sulphate.

All samples were frozen within 30 minutes, moved to −80 °C within 24 hours, and stored there until processing.

### DNA extraction

DNA from the sampling swabs or 125 mg of dam feces was extracted using the ZymoBIOMICS™ DNA Miniprep Kit (Zymo Research, Irvine, CA, USA) according to the manufacturer’s instructions with minor modifications to the bead beating process, as described here. The bead beating was done using a FastPrep® 24 Instrument (MP Biomedicals, Inc., USA) in two successive rounds at 5.5 m/s for 3 minutes. In order to prevent excessive shearing of DNA, the lysate fraction following the first round bead beating was collected and additional ZymoBIOMICS™ Lysis Solution was added before performing the second round. The lysate produced in the second bead beating round was pooled with the previous lysate prior to continuing the procedure. Empty uterine culture swabs (n = 3) were used as negative controls and processed in the same batch with the newborn samples. The other, higher-biomass samples were processed separately to avoid cross-contamination to the newborn samples. The phases involving opening the tubes were performed in laminar flow cabinet, and the workplace, instruments and pipettes were cleaned routinely with 10% bleach. Certified RNase, DNase, DNA and PCR inhibitor free tubes (STARLAB International, Germany) and Nuclease-free Water (Ambion™, Thermo Fisher Scientific, USA) were used for DNA extraction and all downstream analysis. Extracted DNA was stored at −80 °C.

### Quantitative PCR

A universal probe and primers set targeting the bacterial 16S rRNA gene was used for the quantification of 16S rRNA gene copy numbers in the DNA extracted from the calf rectal samples and negative controls, as described in detail in Supplementary information. It was not possible to reliably quantify the amount of meconium and mucosa collected in the sampling swabs, because of the large mass of the intact sampling device. Therefore, we were not able to calculate the absolute quantities of 16S rDNA copies per gram of sampled material. The results are best interpreted as relative copy numbers in the newborn rectal sampling swabs versus the controls and older calf fecal samples.

### Sequencing library preparation and 16S rDNA amplicon sequencing

The hypervariable regions V3 and V4 of the 16S rRNA gene were sequenced using the Illumina MiSeq platform in the DNA core facility of the University of Helsinki, as described in detail in the Supplementary information. The first-round PCR was done using primers 341 F and 785 R. Control and newborn samples were preamplified by 21 PCR cycles, 7-day and adult fecal samples by 15, and others by 18 to compensate for different quantities of 16S rDNA copies in the samples.

### Bioinformatics

The bioinformatics pipeline is described in detail in the Supplementary information. Paired reads were merged to contigs using USEARCH v9.2.64^61^. The contigs were further processed with mothur v. 1.39.5^62^, using Silva v. 128^63^ for alignment and Ribosomal Database Project (RDP) v. 16^64^ for classification. The mothur standard operating procedure for MiSeq data was followed with the exception that potential contaminant genotypes (16S rDNA sequences possibly originating from reagents or instruments) were removed from the data after preclustering and chimera removal. The filtering logic was based on comparing the relative abundances of genotypes in samples and negative controls^29^: genotypes shared with the negative controls were only accepted if their relative abundance in the samples was more than four times as high as in the controls. This type of data decontamination minimizes false positive observations but will delete some taxa which were genuinely present in the samples. Rare OTUs (observed <250 times in the entire dataset) were removed from all samples before data analysis.

### Statistics

The statistical significance of the difference between qPCR measurements in newborns and controls was assessed by the non-parametric two-tailed Mann-Whitney U test, using IBM SPSS Statistics 23. The bacterial alpha diversity was determined as Shannon diversity and as Hill numbers from OTU level data. Shannon diversities between newborn, 24 h and 7 d calves were compared by Friedman rank sum test. Significant differences were then subjected to pairwise comparisons using Nemenyi multiple comparison test with q approximation for unreplicated blocked data, using the R package PMCMR^65^. The principal co-ordinate plots were generated from genus-level taxonomic data, calculating the Bray-Curtis dissimilarities. Similarity between sample types was determined using Spearman (ρ) rank correlation, calculating pairwise correlations from genus-level microbiota composition data between all newborn and older animals. The correlations were compared using Friedman and Nemenyi tests. Venn diagrams were generated using Venny 2.161^66^.

### Data Availability

The original 16S rDNA amplicon sequencing dataset is available in the NCBI SRA database with accession SRP128833.

## Electronic supplementary material

Supplementary information

Dataset 1
