# Publisher Correction: The composition of the perinatal intestinal microbiota in cattle

**DOI:** 10.1038/s41598-018-31494-3

**Published:** 2018-09-11

**Authors:** Mohammad Jaber Alipour, Jonna Jalanka, Tiina Pessa-Morikawa, Tuomo Kokkonen, Reetta Satokari, Ulla Hynönen, Antti Iivanainen, Mikael Niku

**Affiliations:** 1 Department of Veterinary Biosciences, Faculty of Veterinary Medicine, University of Helsinki, Helsinki, Finland; 2 Immunobiology Research Program, Faculty of Medicine, University of Helsinki, Helsinki, Finland; 3 Department of Agricultural Sciences, Faculty of Agriculture and Forestry, University of Helsinki, Helsinki, Finland

Correction to: *Scientific Reports* 10.1038/s41598-018-28733-y, published online 11 July 2018

This original version of this Article contained an error in the order of the Figures. Figures 4, 5 and 7 were published as Figures 7, 4 and 5 respectively. The Figure legends were correct at the time of publication.

The original version of this Article also contained a grayscale image for Figure 4.

These errors have now been corrected in the PDF and HTML versions of the paper.

