# Supplementary material for: The composition of the perinatal intestinal microbiota in cattle
Source: Sci Rep. 2018 Jul 11;8:10437. doi: 10.1038/s41598-018-28733-y (PMC6041309; doi:10.1038/s41598-018-28733-y)
Supplement: Supplementary file 1 — Supplementary information [file 41598_2018_28733_MOESM1_ESM.pdf]

Supplementary information
The composition of the perinatal intestinal microbiota in cattle

Mohammad Jaber Alipour[1], Jonna Jalanka[2], Tiina Pessa-Morikawa[1], Tuomo Kokkonen[3], Reetta Satokari[2], Ulla Hynönen[1], Antti Iivanainen[1*], Mikael Niku[1*]

[1] Department of Veterinary Biosciences, Faculty of Veterinary Medicine, University of Helsinki, Finland
[2] Immunobiology Research Program, Faculty of Medicine, University of Helsinki, Finland
[3] Department of Agricultural Sciences, Faculty of Agriculture and Forestry, University of Helsinki, Finland
* Shared senior authorship

Correspondence to: MN (mikael.niku@helsinki.fi)

Contents

Supplementary results and discussion 2
- Full taxon table 2
- Evaluation of the sequencing data decontamination protocol 2
- Microbial diversity in calves as Hill numbers 5
- Genus-level compositions of dominant phyla 7
- Reoccurrence of newborn core taxa in 24 h and 7 day calves 11
- Characterization of the adult cow microbiota 12

Supplementary methods 15
- Quantitative PCR 15
- MiSeq amplicon sequencing of 16S rDNA 16
- Detailed description of the bioinformatics pipeline 17

Supplementary references 18

Supplementary results and discussion

Full taxon table

Supplementary Table 1 containing all observed taxa is included as a separate Excel spreadsheet file (Supplementary Dataset 1).

Evaluation of the sequencing data decontamination protocol

Impact of data decontamination is shown at genus level in Supplementary Table 2 for newborn data. The protocol completely removed obvious reagent contaminants such as the thermophilic *Hydrogenophilus*, despite its high abundance in the raw data.

Supplementary Table 2. Effect of data decontamination on taxonomic composition of newborn calf meconium samples (raw data vs. decontaminated data). Medians per sample.

**Taxon**	**Median relative abundances**		**Median absolute reads**	
	RAW	***DECONTAMINATED***	***RAW***	***DECONTAMINATED***
Completely deleted taxa				
Hydrogenophilus	18.533 %	0.000 %	4391	0
Escherichia/Shigella	3.432 %	0.000 %	772	0
Schlegelella	3.257 %	0.000 %	728	0
Tepidiphilus	3.459 %	0.000 %	658	0
Enhydrobacter	2.181 %	0.000 %	424	0
Comamonadaceae-unclassified	1.731 %	0.000 %	422	0
Propionibacterium	1.012 %	0.000 %	226	0
Aerococcus	0.776 %	0.000 %	172	0
Anoxybacillus	0.438 %	0.000 %	102	0
Tissierella	0.415 %	0.000 %	89	0
Ralstonia	0.372 %	0.000 %	75	0
Vulcaniibacterium	0.361 %	0.000 %	74	0
Curvibacter	0.181 %	0.000 %	46	0
Xanthomonas	0.186 %	0.000 %	46	0
Cloacibacterium	0.117 %	0.000 %	41	0
Effusibacillus	0.109 %	0.000 %	20	0
Mycobacterium	0.062 %	0.000 %	17	0
Tepidimonas	0.044 %	0.000 %	17	0
Intrasporangiaceae-unclassified	0.077 %	0.000 %	16	0
Haemophilus	0.066 %	0.000 %	13	0
Actinomycetales-unclassified	0.074 %	0.000 %	12	0
Erythrobacteraceae-unclassified	0.056 %	0.000 %	12	0
Aquabacterium	0.042 %	0.000 %	12	0
Anaerococcus	0.064 %	0.000 %	11	0
Arthrobacter	0.020 %	0.000 %	6	0
Faecalibacterium	0.014 %	0.000 %	3	0
Dorea	0.007 %	0.000 %	1	0
Butyricicoccus	0.002 %	0.000 %	1	0
Neisseriaceae-unclassified	0.007 %	0.000 %	1	0
Aeromonas	0.002 %	0.000 %	1	0

Pantoea	0.005 %	0.000 %	1	0
Moraxellaceae-unclassified	0.004 %	0.000 %	1	0
Psychrobacter	0.005 %	0.000 %	1	0

Reduced taxa

Staphylococcus	10.454 %	2.526 %	2441	350
Bacillus	2.296 %	0.199 %	465	14
Atopostipes	0.389 %	0.112 %	77	14
Brevundimonas	0.341 %	0.156 %	77	16
Rheinheimera	0.202 %	0.014 %	56	1
Brevibacterium	0.157 %	0.114 %	36	5
Enterococcus	0.129 %	0.012 %	28	1
Bifidobacterium	0.006 %	0.003 %	1	1

Taxa with increased relative abundance

Acinetobacter	3.877 %	6.562 %	811	495
Corynebacterium	1.660 %	2.793 %	322	263
Streptococcus	1.219 %	2.666 %	245	133
Kocuria	0.745 %	1.019 %	155	46
Pseudomonas	0.666 %	2.733 %	147	147
Massilia	0.564 %	0.679 %	142	77
Micrococcus	0.461 %	0.695 %	121	61
Ruminococcaceae-unclassified	0.502 %	2.166 %	112	111
Paracoccus	0.471 %	0.607 %	93	46
Jeotgalicoccus	0.325 %	0.698 %	67	67
Lactobacillus	0.356 %	0.657 %	64	52
Enterobacteriaceae-unclassified	0.274 %	0.891 %	63	71
Neisseria	0.292 %	0.616 %	60	60
Romboutsia	0.328 %	0.736 %	59	59
Lachnospiraceae-unclassified	0.268 %	1.073 %	54	54
Chryseobacterium	0.291 %	0.320 %	52	22
Clostridium-XI	0.267 %	0.595 %	48	48
Sphingomonas	0.217 %	0.591 %	45	38
Bacteroides	0.251 %	0.565 %	43	22
Bacteroidetes-unclassified	0.180 %	0.390 %	37	32
Clostridiales-unclassified	0.191 %	0.634 %	36	37
Facklamia	0.183 %	0.399 %	33	37
Aerococcaceae-unclassified	0.107 %	0.178 %	28	28
Porphyromonadaceae-unclassified	0.131 %	0.462 %	26	26
Turicibacter	0.134 %	0.525 %	26	25
Pasteurellaceae-unclassified	0.098 %	0.124 %	24	7
Comamonas	0.106 %	0.240 %	24	24
Brachybacterium	0.111 %	0.508 %	20	30
Gemella	0.031 %	0.164 %	17	17
Lactobacillales-unclassified	0.063 %	0.125 %	15	15

Rothia	0.061 %	0.146 %	12	12
Dietzia	0.039 %	0.094 %	11	11
Clostridium-XlVa	0.038 %	0.119 %	9	10
Bacteroidales-unclassified	0.054 %	0.095 %	8	12
Firmicutes-unclassified	0.057 %	0.115 %	8	8
Blastococcus	0.056 %	0.170 %	8	8
Microbacteriaceae-unclassified	0.035 %	0.049 %	7	6
Alistipes	0.036 %	0.095 %	7	7
Rubellimicrobium	0.023 %	0.073 %	7	7
Bacteria-unclassified	0.041 %	0.217 %	7	7
Clostridium-sensu-stricto	0.026 %	0.026 %	5	1
Novosphingobium	0.026 %	0.064 %	5	5
Clostridium-IV	0.016 %	0.049 %	5	3
Rhizobiaceae-unclassified	0.027 %	0.035 %	4	4
Weissella	0.027 %	0.166 %	4	4
Carnobacteriaceae-unclassified	0.006 %	0.012 %	2	1
Moraxella	0.007 %	0.010 %	1	1
Acidovorax	0.000 %	0.112 %	0	14

The data decontamination protocol did not significantly change the phylum-level compositions in adult samples and in the 24h calf rectal samples, compared to uncleaned data (Supplementary Fig. 1). In newborn meconium samples, the proportion of Proteobacteria decreased while Firmicutes, Actinobacteria and Bacteroidetes increased, and in 7-day samples most Proteobacteria were removed. In negative controls, almost all reads were deleted.

In newborn and 7-day samples, a larger number of sequences were deleted than suggested by qPCR quantification of microbial DNA in samples and controls, suggesting that the stringent data decontamination deleted some sequences genuinely originating from the samples.

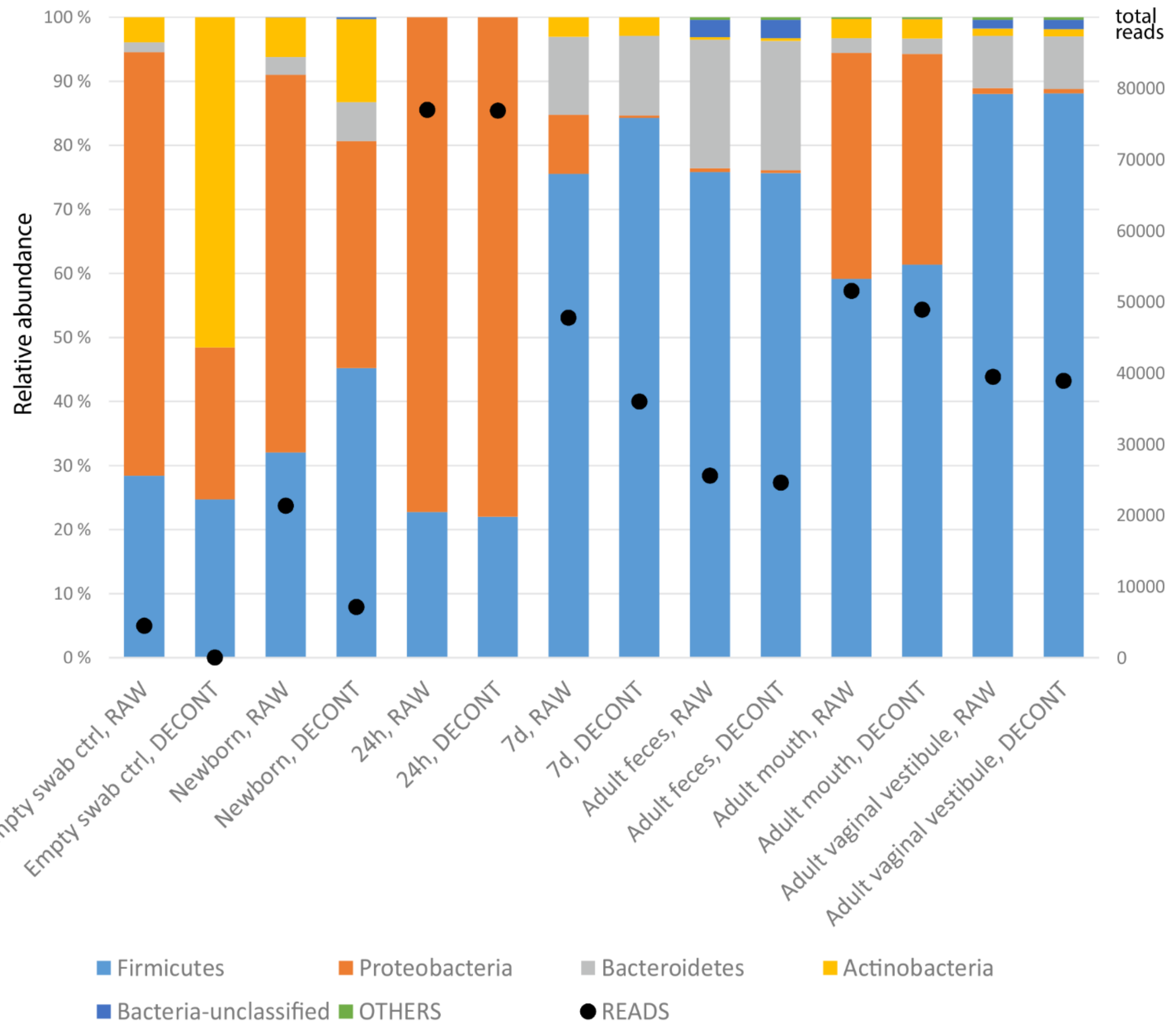

Supplementary Figure 1. Effect of data decontamination on phylum-level compositions (median relative abundances and median absolute sequence read numbers per sample).

Microbial diversity in calves as Hill numbers

Alpha diversity in newborn, 24h and 7d calves as Hill numbers (effective numbers of species) is presented in Supplementary Figure 2A. This measure of diversity facilitates straightforward interpretation and comparison due to its doubling property[1]; however, it has been rarely used in microbiota studies. Most of the common diversity indices can be converted to the effective number of species. This is the number of equally abundant species which produces the given value of the diversity measure used to calculate it[2]. In our case, OTUs represent species.

The various alpha diversity indices can also be represented as a continuous plot, as a function of *q*[3-6], which gives a more complete understanding of the diversity (Fig. S2B):

$$Hill\ number = \left(\sum_{i=1}^{S} p_i^q\right)^{1/(1-q)}, q \geq 0, q \neq 1$$

where *S* = the number of species (in our case, OTUs), and p_i = the relative abundance of each species (OTU); $p_i > 0$ and $\sum_{i=1}^{S} p_i = 1$. The parameter *q* determines the sensitivity of an index to the common and rare species[5-7]. At *q*=0, the Hill number is simply the observed species richness. The higher *q* values emphasize the abundant species[1,7,8]. At *q*=1, the function is undefined, and therefore the estimate $e^{-\Sigma(p \times \ln(p))}$is

applied; this is the exponential of the Shannon diversity index. When *q* approaches infinity, the Hill number approaches the inverse of the maximum of *p*, thus representing only the most abundant species.

In our data, all the diversity measures showed that the alpha diversity collapsed after birth, then started to increase by 7 days of age in most calves. The dominance of abundant species (OTUs) was greatest in the 24 h samples, as richness was proportionally higher than the other indices.

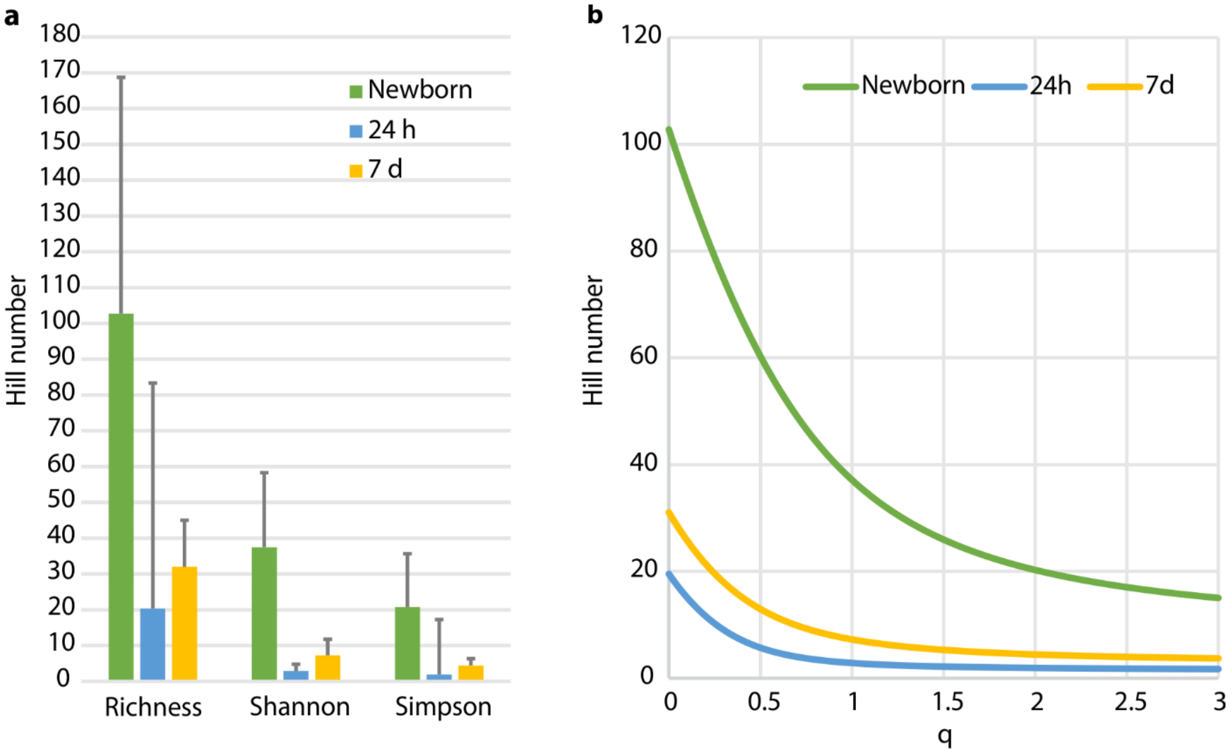

Supplementary Figure 2. Alpha diversity in calf rectum as Hill numbers (effective number of OTUs). *A) Observed richness (q=0), Shannon effective number of OTUs (q=1; calculated as $e^{(Shannon\ diversity\ index)}$) and Simpson effective number of OTUs [q=2; calculated as 1/(Simpson diversity index)]. Averages and standard deviations are shown. B) Continuous plots of Hill numbers as function of q.*

Genus-level compositions of dominant phyla

Genus-level compositions and alpha diversities within major phyla are shown in Supplementary Figs. 3-6. The relatively high diversity and similarity to adult oral microbiota is apparent in newborns also here.

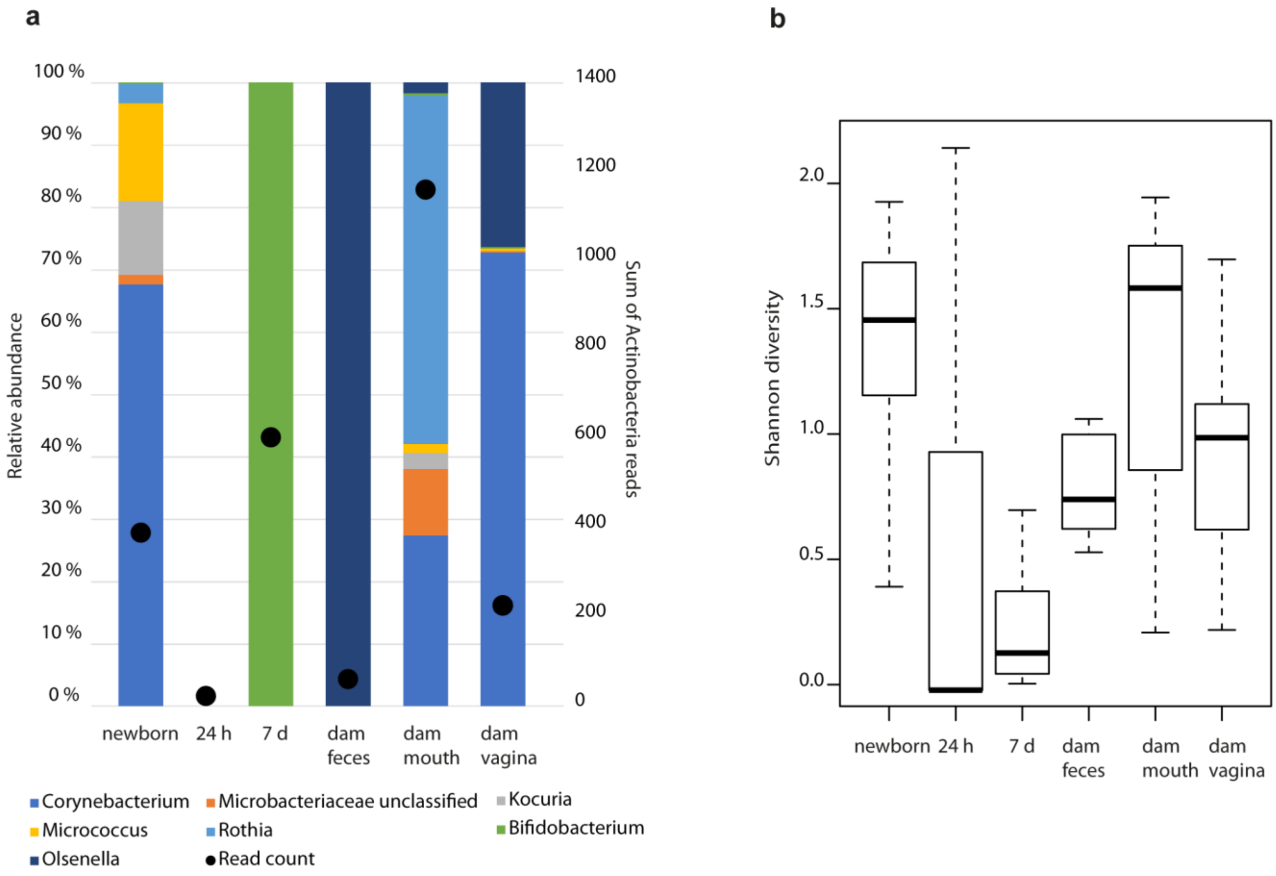

Supplementary Figure 3. *Genus-level composition (A) and Shannon diversity (B) within Actinobacteria. The compositions and read counts are medians, and therefore some sample groups appear as zero reads, although individual samples may contain data. The Shannon diversity presented here was calculated from genus-level data.*

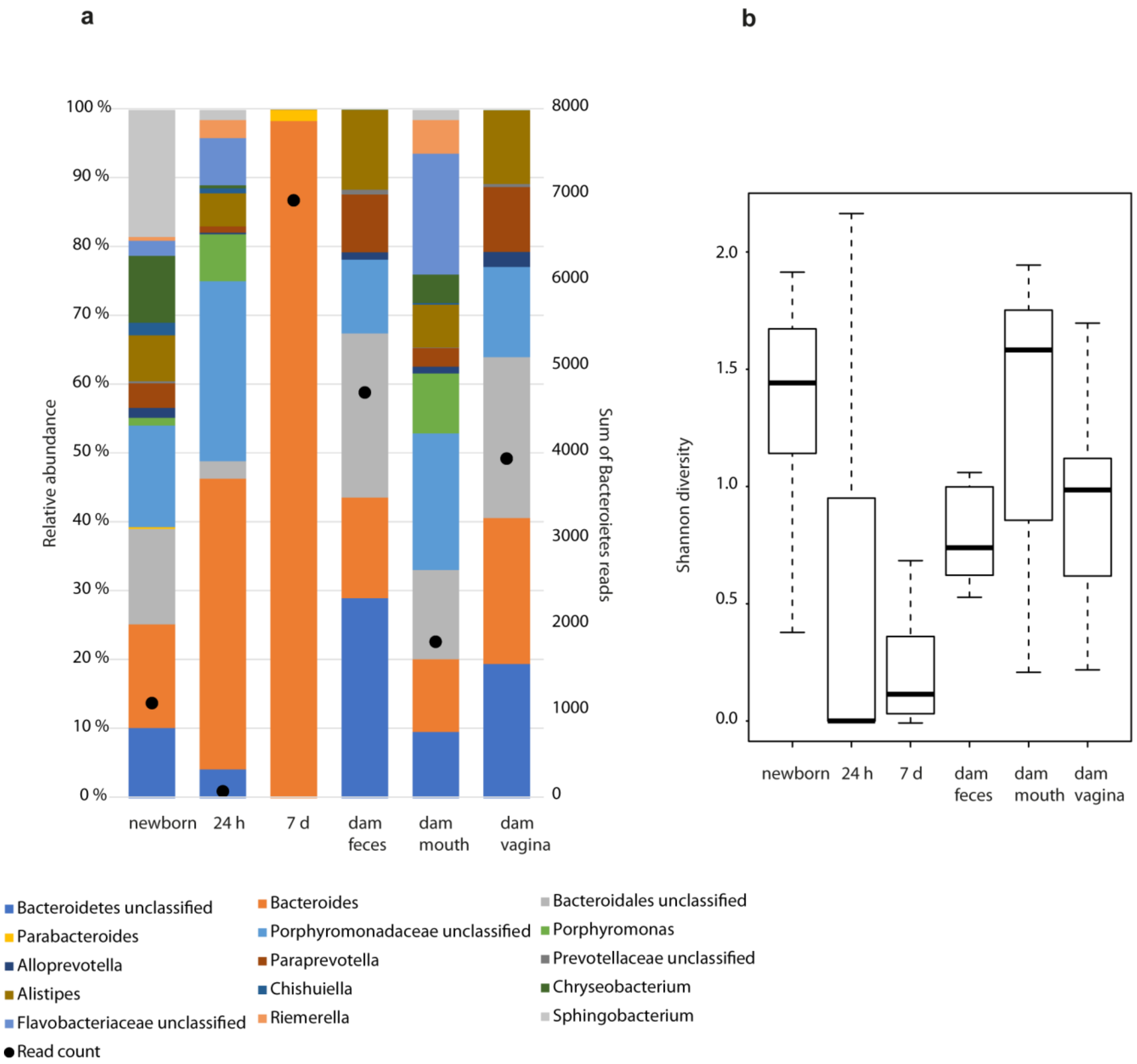

Supplementary Figure 4. *Genus-level composition (A) and Shannon diversity (B) within Bacteroidetes. The compositions and read counts are medians, and therefore some sample groups appear as zero reads, although individual samples may contain data. The Shannon diversity presented here was calculated from genus-level data.*

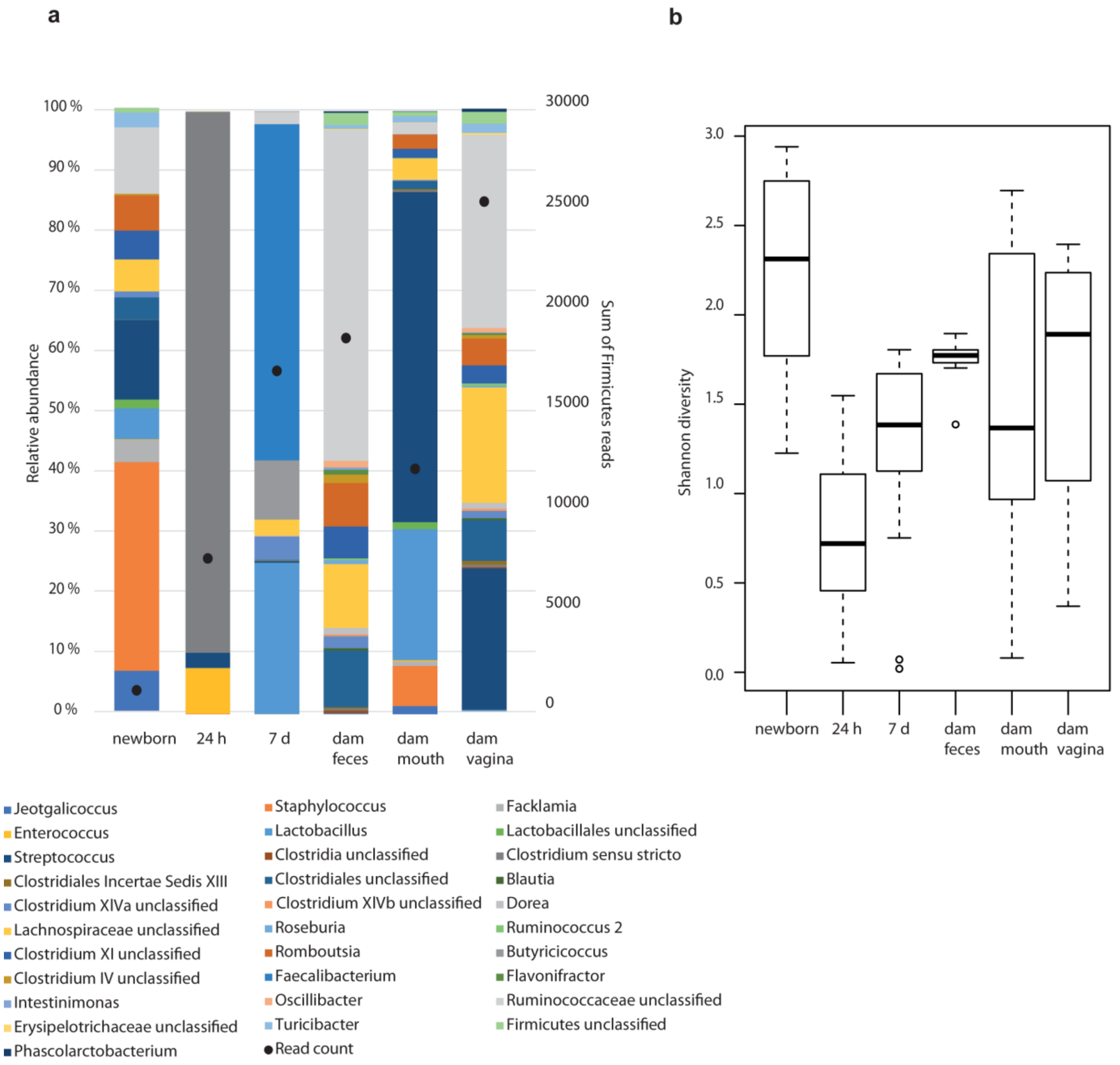

Supplementary Figure 5. *Genus-level composition (A) and Shannon diversity (B) within Firmicutes. The compositions and read counts are medians. The Shannon diversity presented here was calculated from genus-level data.*

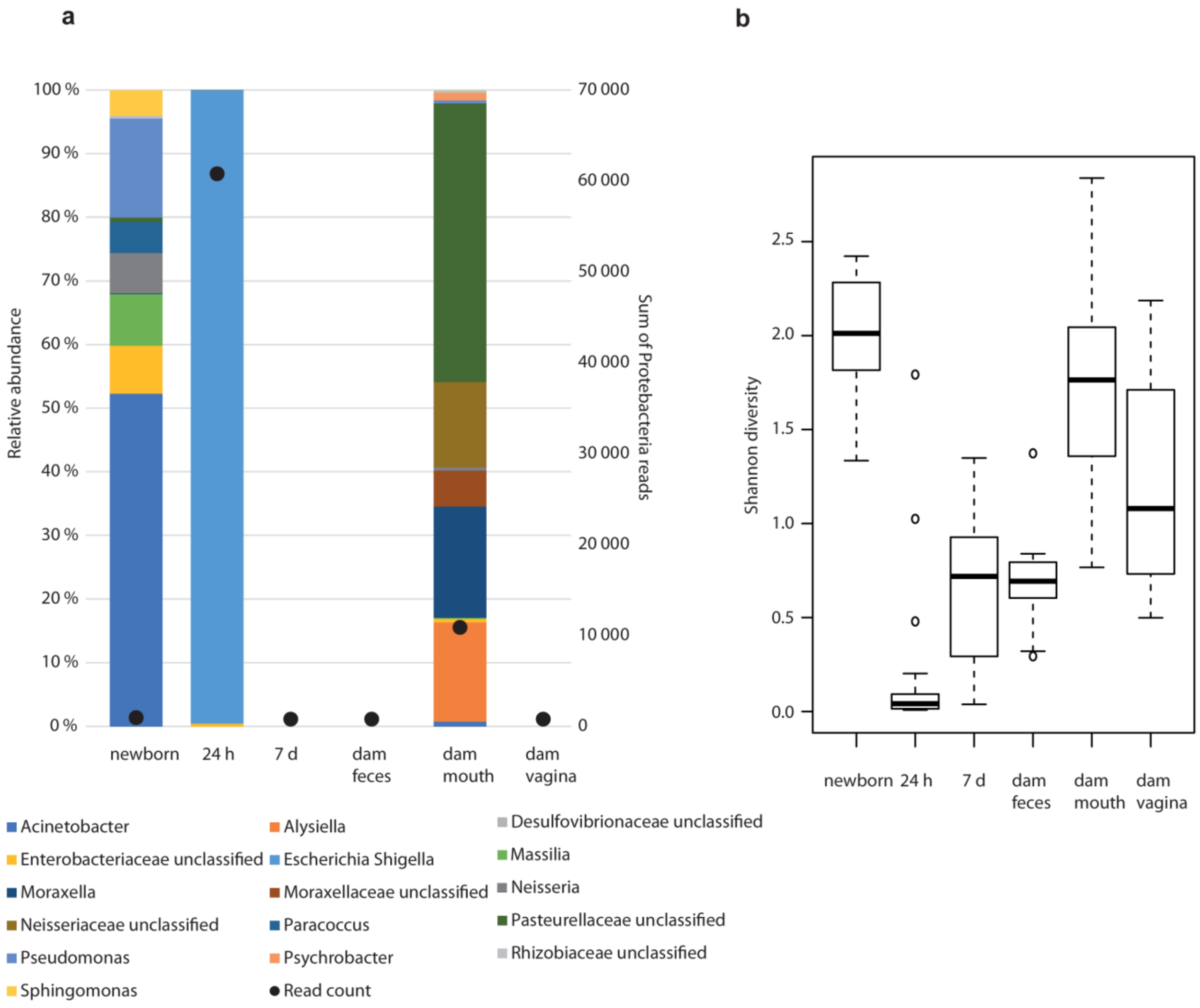

Supplementary Figure 6. *Genus-level composition (A) and Shannon diversity (B) within Proteobacteria. The compositions and read counts are medians, and therefore some sample groups appear as zero reads, although individual samples may contain data. The Shannon diversity presented here was calculated from genus-level data.*

Reoccurrence of newborn core taxa in 24 h and 7 day calves

Supplementary Table 3 shows the occurrence of newborn core taxa in older calves. Most Actinobacteria and Proteobacteria were no longer detectable in most of the older calves, while Firmicutes and *Bacteroides* largely remained.

Supplementary Table 3: Prevalence of newborn core bacterial taxa in newborns and older calves. *"Reoccurrence" shows prevalence in 24 h and 7 d calves which were positive at birth. "Appearance" shows prevalence in calves which were negative at birth. A calf was considered positive if the taxon was detectable, even at a low level. NB = newborn.*

		Reoccurrence		***Appearance***	
**Taxon**	**Newborns**	**NB vs 24h**	**NB vs 7d**	**24h vs NB**	**7d vs NB**
**Actinobacteria**					
Corynebacterium	100 %	48 %	24 %		
Brachybacterium	95 %	10 %	0 %	0 %	0 %
Kocuria	95 %	33 %	10 %	0 %	0 %
Micrococcus	86 %	14 %	0 %	0 %	0 %
Rothia	86 %	38 %	6 %	33 %	0 %
**Bacteroidetes**					
Unclassified *Bacteroidetes*	76 %	10 %	6 %	20 %	0 %
Bacteroides	90 %	57 %	100 %	50 %	100 %
**Firmicutes**					
Staphylococcus	100 %	86 %	62 %		
Lactobacillus	95 %	90 %	100 %	100 %	100 %
Streptococcus	95 %	90 %	95 %	100 %	100 %
Unclassified *Clostridiales*	76 %	38 %	38 %	20 %	60 %
Clostridium-XlVa	76 %	43 %	88 %	40 %	100 %
Unclassified *Lachnospiraceae*	95 %	76 %	90 %	100 %	100 %
Clostridium-XI	76 %	38 %	13 %	60 %	20 %
Romboutsia	76 %	52 %	50 %	60 %	40 %
Unclassified *Ruminococcaceae*	95 %	90 %	95 %	100 %	100 %
**Proteobacteria**					
Brevundimonas	86 %	5 %	0 %	0 %	0 %
Paracoccus	86 %	5 %	0 %	0 %	0 %
Sphingomonas	100 %	5 %	0 %		
Comamonas	76 %	10 %	19 %	0 %	20 %
Massilia	90 %	14 %	16 %	0 %	0 %
Neisseria	90 %	14 %	16 %	0 %	0 %
Unclassified *Enterobacteriaceae*	95 %	86 %	45 %	100 %	100 %
Unclassified *Pasteurellaceae*	81 %	38 %	29 %	75 %	100 %
Acinetobacter	100 %	38 %	0 %		
Pseudomonas	95 %	24 %	0 %	0 %	0 %

Characterization of the adult cow microbiota

The core genera in adult cows are shown in Supplementary Tables 4-6.

The adult fecal microbiota was composed primarily of Firmicutes and Bacteroidetes. 41% of all sequence reads represented unclassified genera of the *Ruminococcaceae* family (Supplementary Table 4). Unclassified *Lachnospiraceae* (8%), unclassified *Clostridiales* (7%), unclassified *Bacteroidetes* (5%), *Romboutsia* (5%), unclassified *Bacteroidales* (4%), *Clostridium* cluster XI (4%), *Bacteroides* (3%) and *Alistipes* (2%) were also abundant. All the core taxa (at >0.1% median abundance) were shared by all 10 cows. The inter-individual variation was very small in terms of alpha and beta diversity (see figures included in the article), probably due to the similarity in housing and feeding. The phylum-level composition of bovine fecal microbiota has been shown to be greatly affected by feeding, especially by the starch content of the diet[9]. The cows in our study were fed grass silage, supplemented with a small amount of commercial concentrate (mainly barley, wheat and rape seed meal) for about 1-2 weeks before sampling.

The cow oral microbiota was mostly composed of Firmicutes (62%) and Proteobacteria (33%), with smaller contributions of Actinobacteria and Bacteroidetes. The most abundant taxa included *Streptococcus* (12%), *Lactobacillus* (7%), unclassified *Pasteurellaceae* (7%), *Alysiella* (3%), *Moraxella* (3%), and *Staphylococcus* (2%), also shared by all the cows (Supplementary Table 5). The oral microbiota observed in our study was more similar to human oral microbiota than to the recently reported bovine oral microbiota sampled at the time of ingesta regurgitation, possibly because we did not collect the samples at regurgitation[10,11].

The dam reproductive tract microbiota was sampled at the cranial vaginal vestibule, close to the opening of the urethra, rather than deep in the vagina, to avoid risk to the pregnancy. The microbiota of the vaginal vestibule microbiota was mostly composed of Firmicutes, with <8% Bacteroidetes. Unclassified *Ruminococcaceae* (21%), *Streptococcus* (16%), unclassified *Lachnospiraceae* (10%), unclassified *Clostridiales* (4%), *Romboutsia* (3%), *Bacteroides* (2%) and *Clostridium* XI were the most abundant taxa (Supplementary Table 6). All the core taxa were shared by all 10 cows. The vestibular microbiota was very similar to the fecal microbiota (see figures included in the article). However, *Streptococcus* was much more numerous in the vestibule than in feces, and *Corynebacterium*, unclassified members of Candidatus Saccharibacteria and *Aerococcus* were present in all vaginal samples but not detected in the feces of any cow. *Lactobacillus* was found in the vestibule of all cows but at <0.1% median abundance, as described previously for cow vaginal microbiota[12]. The phylum-level composition in our data differs from the few previously published studies of cow vaginal microbiota probably because of differences in sampling (mucosal swabs versus lavage) and because the vaginal microbiota changes during pregnancy[13].

Supplementary Table 4: Core bacterial taxa (>0.1% relative abundance, >75% prevalence) in adult cow feces. *Median relative abundances and prevalence in 10 cows are shown.*

Taxon	Abundance	SD	Prevalence
Firmicutes			
Clostridia; Clostridiales; Clostridiaceae-1; Clostridium-sensu-stricto	*0.3 %*	*0.8 %*	*100 %*
Clostridia; Clostridiales; Lachnospiraceae; Blautia	*0.3 %*	*0.1 %*	*100 %*
Clostridia; Clostridiales; Lachnospiraceae; Clostridium-XlVa	*1.5 %*	*0.6 %*	*100 %*
Clostridia; Clostridiales; Lachnospiraceae; Clostridium-XlVb	*0.2 %*	*0.1 %*	*100 %*
Clostridia; Clostridiales; Lachnospiraceae; Dorea	*0.8 %*	*0.4 %*	*100 %*
Clostridia; Clostridiales; Lachnospiraceae; Roseburia	*0.5 %*	*0.3 %*	*100 %*
Clostridia; Clostridiales; Lachnospiraceae; Ruminococcus2	*0.2 %*	*0.1 %*	*100 %*
Clostridia; Clostridiales; Peptococcaceae-1; Peptococcus	*0.1 %*	*0.1 %*	*100 %*
Clostridia; Clostridiales; Peptostreptococcaceae; Clostridium-XI	*3.8 %*	*2.0 %*	*100 %*

Clostridia; Clostridiales; Peptostreptococcaceae; Romboutsia	*5.1 %*	*3.3 %*	*100 %*
Clostridia; Clostridiales; Ruminococcaceae; Clostridium-IV	*1.1 %*	*0.4 %*	*100 %*
Clostridia; Clostridiales; Ruminococcaceae; Flavonifractor	*0.6 %*	*0.2 %*	*100 %*
Clostridia; Clostridiales; Ruminococcaceae; Intestinimonas	*0.3 %*	*0.4 %*	*100 %*
Clostridia; Clostridiales; Ruminococcaceae; Oscillibacter	*0.8 %*	*0.2 %*	*100 %*
Clostridia; Clostridiales; unclassified *Clostridiales-Incertae-Sedis-XIII*	*0.1 %*	*0.1 %*	*100 %*
Clostridia; Clostridiales; unclassified *Lachnospiraceae*	*8.1 %*	*5.2 %*	*100 %*
Clostridia; Clostridiales; unclassified *Ruminococcaceae*	*40.9 %*	*3.9 %*	*100 %*
Clostridia; unclassified Clostridiales	*7.3 %*	*2.9 %*	*100 %*
Erysipelotrichia; Erysipelotrichales; Erysipelotrichaceae; Turicibacter	*0.5 %*	*0.7 %*	*100 %*
Erysipelotrichia; Erysipelotrichales; unclassified *Erysipelotrichaceae*	*0.1 %*	*0.1 %*	*100 %*
Negativicutes; Selenomonadales; Acidaminococcaceae; Phascolarctobacterium	*0.2 %*	*0.0 %*	*100 %*
Unclassified *Clostridia*	*0.4 %*	*0.3 %*	*100 %*
Unclassified *Firmicutes*	*1.6 %*	*0.9 %*	*100 %*
Bacteroidetes			
Bacteroidia; Bacteroidales; Bacteroidaceae; Bacteroides	*2.9 %*	*1.4 %*	*100 %*
Bacteroidia; Bacteroidales; Prevotellaceae; Alloprevotella	*0.1 %*	*0.1 %*	*100 %*
Bacteroidia; Bacteroidales; Prevotellaceae; Paraprevotella	*1.2 %*	*1.8 %*	*100 %*
Bacteroidia; Bacteroidales; Prevotellaceae; Prevotella	*0.1 %*	*0.1 %*	*100 %*
Bacteroidia; Bacteroidales; Rikenellaceae; Alistipes	*2.0 %*	*0.8 %*	*100 %*
Bacteroidia; Bacteroidales; unclassified *Porphyromonadaceae*	*1.7 %*	*0.8 %*	*100 %*
Bacteroidia; unclassified *Bacteroidales*	*4.4 %*	*1.0 %*	*100 %*
Unclassified *Bacteroidetes*	*5.3 %*	*1.5 %*	*100 %*
Proteobacteria			
Deltaproteobacteria; Desulfovibrionales; unclassified *Desulfovibrionaceae;*	*0.3 %*	*0.4 %*	*100 %*
Actinobacteria			
Actinobacteria; Coriobacteriales; Coriobacteriaceae; Olsenella	*0.2 %*	*0.1 %*	*100 %*
Verrucomicrobia			
Verrucomicrobiae; Verrucomicrobiales; Verrucomicrobiaceae; Akkermansia	*0.3 %*	*0.2 %*	*100 %*
Unclassified bacteria	2.9 %	1.4 %	100 %

Supplementary Table 5: Core bacterial taxa (>0.1% relative abundance, >75% prevalence) in adult cow mouth. *Median relative abundances and prevalence in 10 cows are shown.*

**Taxon**	**Abundance**	**SD**	**Prevalence**
Firmicutes			
Bacilli; Bacillales; Staphylococcaceae; Jeotgalicoccus	*0.4 %*	*1.0 %*	*90 %*
Bacilli; Bacillales; Staphylococcaceae; Staphylococcus	*2.1 %*	*16.7 %*	*100 %*
Bacilli; Lactobacillales; Aerococcaceae; Facklamia	*0.2 %*	*1.9 %*	*90 %*
Bacilli; Lactobacillales; Lactobacillaceae; Lactobacillus	*7.0 %*	*18.5 %*	*100 %*
Bacilli; Lactobacillales; Lactobacillaceae; Pediococcus	*0.1 %*	*0.4 %*	*80 %*
Bacilli; Lactobacillales; Streptococcaceae; Streptococcus	*12.3 %*	*22.0 %*	*100 %*

Bacilli; Lactobacillales; unclassified *Carnobacteriaceae*	*0.2 %*	*0.2 %*	*90 %*
Bacilli; unclassified *Lactobacillales*	*0.3 %*	*0.8 %*	*100 %*
Clostridia; Clostridiales; Peptostreptococcaceae; Clostridium-XI	*0.3 %*	*1.9 %*	*100 %*
Clostridia; Clostridiales; Peptostreptococcaceae; Romboutsia	*0.5 %*	*2.6 %*	*100 %*
Clostridia; Clostridiales; unclassified *Lachnospiraceae*	*0.7 %*	*3.1 %*	*100 %*
Clostridia; Clostridiales; unclassified *Ruminococcaceae*	*0.4 %*	*7.4 %*	*100 %*
Clostridia; unclassified *Clostridiales*	*0.3 %*	*1.4 %*	*90 %*
Erysipelotrichia; Erysipelotrichales; Erysipelotrichaceae; Turicibacter	*0.2 %*	*1.1 %*	*100 %*
Unclassified *Firmicutes*	*0.2 %*	*0.4 %*	*100 %*
Proteobacteria			
Alphaproteobacteria; Rhizobiales; unclassified *Rhizobiaceae*	*0.1 %*	*0.2 %*	*90 %*
Betaproteobacteria; Neisseriales; Neisseriaceae; Alysiella	*3.0 %*	*3.9 %*	*100 %*
Betaproteobacteria; Neisseriales; Neisseriaceae; Neisseria	*0.1 %*	*2.6 %*	*100 %*
Betaproteobacteria; Neisseriales; unclassified *Neisseriaceae*	*2.6 %*	*2.1 %*	*90 %*
Gammaproteobacteria; Enterobacteriales; unclassified *Enterobacteriaceae*	*0.1 %*	*2.5 %*	*90 %*
Gammaproteobacteria; Pasteurellales; unclassified *Pasteurellaceae*	*6.5 %*	*10.3 %*	*100 %*
Gammaproteobacteria; Pseudomonadales; Moraxellaceae; Acinetobacter	*0.1 %*	*0.2 %*	*90 %*
Gammaproteobacteria; Pseudomonadales; Moraxellaceae; Moraxella	*3.0 %*	*3.7 %*	*100 %*
Gammaproteobacteria; Pseudomonadales; Moraxellaceae; Psychrobacter	*0.5 %*	*0.7 %*	*90 %*
Gammaproteobacteria; Pseudomonadales; unclassified *Moraxellaceae*	*1.2 %*	*0.9 %*	*100 %*
Actinobacteria			
Actinobacteria; Actinomycetales; Corynebacteriaceae; Corynebacterium	*0.9 %*	*2.3 %*	*100 %*
Actinobacteria; Actinomycetales; Micrococcaceae; Rothia	*1.0 %*	*0.9 %*	*100 %*
Bacteroidetes			
Bacteroidia; Bacteroidales; Porphyromonadaceae; Porphyromonas	*0.2 %*	*0.3 %*	*90 %*
Bacteroidia; Bacteroidales; unclassified *Porphyromonadaceae*	*0.4 %*	*0.7 %*	*100 %*
Flavobacteriia; Flavobacteriales; unclassified *Flavobacteriaceae*	*0.5 %*	*0.4 %*	*100 %*
Fusobacteria			
Fusobacteriia; Fusobacteriales; unclassified *Leptotrichiaceae*	*0.2 %*	*0.2 %*	*100 %*

Supplementary Table 6: Core bacterial taxa (>0.1% relative abundance, >75% prevalence) in adult cow vaginal vestibule. *Median relative abundances and prevalence in 10 cows are shown.*

**Taxon**	**Abundance**	**SD**	**Prevalence**
Firmicutes			
Bacilli; Lactobacillales; Streptococcaceae; Streptococcus	*15.7 %*	*32.9 %*	*100 %*
Clostridia; Clostridiales; Clostridiaceae-1; Clostridium-sensu-stricto	*0.2 %*	*1.1 %*	*100 %*
Clostridia; Clostridiales; Lachnospiraceae; Blautia	*0.2 %*	*0.1 %*	*100 %*
Clostridia; Clostridiales; Lachnospiraceae; Clostridium-XlVa	*0.9 %*	*0.7 %*	*100 %*
Clostridia; Clostridiales; Lachnospiraceae; Clostridium-XlVb	*0.2 %*	*0.2 %*	*100 %*
Clostridia; Clostridiales; Lachnospiraceae; Dorea	*0.6 %*	*0.5 %*	*100 %*
Clostridia; Clostridiales; Lachnospiraceae; Roseburia	*0.2 %*	*0.1 %*	*100 %*

Clostridia; Clostridiales; Lachnospiraceae; Ruminococcus2	*0.3 %*	*0.2 %*	*100 %*
Clostridia; Clostridiales; Peptostreptococcaceae; Clostridium-XI	*2.2 %*	*2.2 %*	*100 %*
Clostridia; Clostridiales; Peptostreptococcaceae; Romboutsia	*3.3 %*	*3.7 %*	*100 %*
Clostridia; Clostridiales; Ruminococcaceae; Clostridium-IV	*0.4 %*	*0.2 %*	*100 %*
Clostridia; Clostridiales; Ruminococcaceae; Flavonifractor	*0.2 %*	*0.2 %*	*100 %*
Clostridia; Clostridiales; Ruminococcaceae; Oscillibacter	*0.5 %*	*0.3 %*	*100 %*
Clostridia; Clostridiales; unclassified *Clostridiales-Incertae-Sedis-XIII*	*0.4 %*	*0.4 %*	*100 %*
Clostridia; Clostridiales; unclassified *Lachnospiraceae*	*10.4 %*	*7.1 %*	*100 %*
Clostridia; Clostridiales; unclassified *Ruminococcaceae*	*21.4 %*	*13.5 %*	*100 %*
Clostridia; unclassified *Clostridiales*	*4.4 %*	*3.0 %*	*100 %*
Erysipelotrichia; Erysipelotrichales; Erysipelotrichaceae; Turicibacter	*0.8 %*	*2.3 %*	*100 %*
Erysipelotrichia; Erysipelotrichales; unclassified *Erysipelotrichaceae*	*0.2 %*	*0.2 %*	*100 %*
Negativicutes; Selenomonadales; Acidaminococcaceae; Phascolarctobacterium	*0.4 %*	*0.2 %*	*100 %*
Unclassified *Clostridia*	*0.1 %*	*0.2 %*	*100 %*
Unclassified *Firmicutes*	*1.2 %*	*0.8 %*	*100 %*
Bacteroidetes			
Bacteroidia; Bacteroidales; Bacteroidaceae; Bacteroides	*2.3 %*	*1.4 %*	*100 %*
Bacteroidia; Bacteroidales; Prevotellaceae; Alloprevotella	*0.2 %*	*0.2 %*	*100 %*
Bacteroidia; Bacteroidales; Prevotellaceae; Paraprevotella	*0.3 %*	*1.7 %*	*100 %*
Bacteroidia; Bacteroidales; Rikenellaceae; Alistipes	*0.9 %*	*1.0 %*	*100 %*
Bacteroidia; Bacteroidales; unclassified *Porphyromonadaceae*	*0.9 %*	*1.2 %*	*100 %*
Bacteroidia; unclassified *Bacteroidales*	*1.4 %*	*2.3 %*	*100 %*
Unclassified *Bacteroidetes*	*1.6 %*	*1.7 %*	*100 %*
Actinobacteria			
Actinobacteria; Actinomycetales; Corynebacteriaceae; Corynebacterium	*0.4 %*	*3.0 %*	*100 %*
Actinobacteria; Coriobacteriales; Coriobacteriaceae; Olsenella	*0.2 %*	*0.3 %*	*100 %*
Proteobacteria			
Deltaproteobacteria; Desulfovibrionales; unclassified *Desulfovibrionaceae*	*0.1 %*	*0.2 %*	*100 %*
Candidatus-Saccharibacteria			
Unclassified *Candidatus-Saccharibacteria*	*0.2 %*	*0.2 %*	*100 %*
Verrucomicrobia			
Verrucomicrobiae; Verrucomicrobiales; Verrucomicrobiaceae; Akkermansia	*0.1 %*	*0.1 %*	*100 %*
Unclassified bacteria	*1.4 %*	*0.8 %*	*100 %*

Supplementary methods

Quantitative PCR

A universal probe and primer set targeting the bacterial 16S rRNA gene was used for the quantification of 16S rRNA gene copy numbers in the DNA extracted from the calf rectal samples and negative controls [forward primer: 5′-TCCTACGGGAGGCAGCAGT-3′; reverse primer: 5′-GGACTACCAGGGTATCTAATCCTGTT-3′;

probe: (6-FAM)-5′-CGTATTACCGCGGCTGCTGGCAC-3′-(BHQ1)][14]. To prepare a standard curve for absolute quantification, the complete 16S rRNA gene from *Lactobacillus amylovorus* (strain GRL1112) was amplified using primers 5′- AGAGTTTGATCCTGGCTCAG-3′ and 5′-ACGGCTACCTTGTTACGACTT-3′[15] . A standard series consisting of seven ten-fold dilutions from $1 \cdot 10^2$ to $1 \cdot 10^8$ copies was used. The qPCR was performed with an Mx3005P instrument (Agilent Technologies, Santa Clara, CA, USA) using DNA free 96 well plates (Eppendorf, Germany). The PCR reaction (25 µl total volume) containing 200 nM of probe and 300 nM of each primer was performed utilizing the ROX-containing 5× HOT FIREPol Probe qPCR Mix Plus (Solis BioDyne, Estonia). All samples were amplified in triplicate by the following thermal cycling conditions: 95°C for 15 min (polymerase activation and initial template denaturation), followed by 40 cycles of 95°C for 15 s (denaturation) and 60°C for 60 s (annealing/elongation). To fit all samples within the dynamic range of qPCR, 2.5 µl of control and newborn DNA extract and 0.05 µl of 24 h and 7 d DNA extracts were used per reaction. Fecal and especially meconial samples contain high concentrations of PCR inhibitors, which are not completely removed by DNA extraction kits. Smaller PCR inhibitor concentrations in the more diluted samples may have allowed more efficient PCR amplification, resulting in values proportionally up to ~50% higher in the 24 h and 7 d samples, based on dilution series tests. The MxPro – Mx3000P software version 4.10 (Agilent Technologies) was used for data analysis.

MiSeq amplicon sequencing of 16S rDNA

The hypervariable regions V3 and V4 of the 16S rRNA gene were sequenced using the Illumina MiSeq platform in the DNA core facility of the University of Helsinki. The library preparation and sequencing were carried out essentially as described previously[16]. The target regions were first amplified by two rounds of PCR. In the first round PCR, each sample was amplified in triplicate in 25 µl total volume, using 1× Phusion Hot Start II High-Fidelity PCR Master Mix (Thermo Scientific), 2.5% DMSO, 500 nM of a mixture of 4 forward and 4 reverse universal bacterial primers (Supplementary Table 5), and 2.5 µl of DNA extracted from each sample (concentrations of the DNA extracts for each sample group as medians ± SD: newborns, 1.1±13 ng/µl; 24h calves, 40±19 ng/µl; 7d calves, 56±48 ng/µl; dam feces, 119±75 ng/µl; dam mouth, 0.63±2.2 ng/µl; dam vaginal vestibule, 20±20 ng/µl). Each sample type was run in a separate batch, with the negative control swabs processed in the same batch with the newborn samples. In every batch, a PCR blank with no template DNA was included. The following thermal cycling conditions were implemented for a 3-step PCR using a T100™ Thermal Cycler (Bio-Rad Laboratories): 98°C for 30 s (polymerase activation and initial denaturation), followed by cycles of 98°C for 10 s, 56°C for 30 s, 72°C for 20 s, and finally 72°C for 5 min. The cycle numbers were optimized based on the template abundance in each sample type, in order to minimize PCR bias: negative controls and newborn calf samples 21 cycles; dam vaginal and buccal samples and 24 h calf fecal samples 18 cycles; and dam fecal samples and 7 d calf fecal samples 15 cycles. The triplicate products of the first round PCR for each sample were pooled, purified with ExoSAP-IT™ (Thermo Fisher Scientific) and used as a template for the second round PCR. The second round PCR amplifications were performed using an Illumina forward and reverse primer set (Supplementary Table 5), Phusion Hot-Start II polymerase (Finnzymes/Thermo Scientific), High Fidelity buffer and 2.5% DMSO. The following thermal cycling conditions were applied with an Arktik thermal cycler (Finnzymes/Thermo Scientific): initial denaturation at 98 °C for 30 s, 17 cycles at 98 °C for 10s, 65°C for 30s, 72 °C for 10s, and a final extension at 72 °C for 5 min. The second round PCR products were pooled in equal amounts, purified with Agencourt AMPure XP magnetic beads (Beckman Coulter) and size selected (500-700 bp) using BluePippin™ (Sage Science, USA). The quantity and quality of the amplicons were assessed with Qubit (Invitrogen, Thermo Scientific) and Bioanalyzer 2100 (Agilent Technologies), respectively. The final 16s rRNA gene amplicons obtained from samples, negative controls and PCR blanks were sequenced on an Illumina MiSeq sequencer using the v2 600 cycle kit paired-end (325 bp + 285 bp).

Supplementary Table 5. Sequencing primers

1st round	FW 5'-3' (341F)	ATCTACACTCTTTCCCTACACGACGCTCTTCCGATCT*CCTACGGGNGGCWGCAG*<br>ATCTACACTCTTTCCCTACACGACGCTCTTCCGATCTgt*CCTACGGGNGGCWGCAG*<br>ATCTACACTCTTTCCCTACACGACGCTCTTCCGATCTagag*CCTACGGGNGGCWGCAG*<br>ATCTACACTCTTTCCCTACACGACGCTCTTCCGATCTtagtgt*CCTACGGGNGGCWGCAG*
	REV 5'-3' (785R)	GTGACTGGAGTTCAGACGTGTGCTCTTCCGATCT*GACTACHVGGGTATCTAATCC*<br>GTGACTGGAGTTCAGACGTGTGCTCTTCCGATCTa*GACTACHVGGGTATCTAATCC*<br>GTGACTGGAGTTCAGACGTGTGCTCTTCCGATCTtct*GACTACHVGGGTATCTAATCC*<br>GTGACTGGAGTTCAGACGTGTGCTCTTCCGATCTctgagtg*GACTACHVGGGTATCTAATCC*
2nd round	FW 5'-3'	AATGATACGGCGACCACCGAGATCTACACTCTTTCCCTACACGAC
	REV 5'-3'	CAAGCAGAAGACGGCATACGAGATXXXXXXXXGTGACTGGAGTTCAGACGTGT

▶**Sequencing primer** ▶*16S specific primer* ▶**Spacer nucleotide** ▶Illumina adapter ▶Inde**X**

Detailed description of the bioinformatics pipeline

Removing primers and spacers

A subsample of the sequence data was first visualized and checked in Geneious[17]. Primers and spacers were removed using CutAdapt[18]. The results were again confirmed in Geneious.

Merging paired reads and quality filtering

Paired reads were merged and quality filtered using USEARCH v.9.2.64[19,20].

Initial pre-processing in mothur

The group file needed for mothur was generated from the USEARCH outputs using unix commands awk and sed. Then, pre-processing was continued using mothur v. 1.39.5, according to mothur MiSeq standard operating procedure (SOP)[21,22], up to de-noising (preclustering) and removal of chimeras and non-bacterial sequences. Silva v. 128[23] was used for alignment and Ribosomal Database Project (RDP) v. 16[24] for classification.

Filtering 16S genotypes shared between samples and negative controls

Genotypes (16S rDNA sequences) which were abundant in negative controls were removed as potential contaminants, after de-noising and removal of chimeras and non-bacterial sequences. This was done for each sample type separately (newborns, 24h, 7d, adult feces, adult oral, adult vulvar vestibule).

First, sequence count tables for each sample type (+ negative controls) were extracted using mothur. For example, for newborn samples:

```
### Here, the .accnosgroups file specifies the samples to be output
get.groups(fasta=calf.nb.fasta, count=calf.nb.count_table, accnos=nb_ns.accnosgroups)
```

These subsets were then processed individually in Excel, to identify potential contaminant genotypes in each sample type. Singletons (only 1 hit in the extracted dataset) were first removed. Then, potential contaminant genotypes were identified based on their relative abundances in negative controls and actual samples, as actual contaminants are expected to be relatively more abundant in the controls[25]. Here, we approved genotypes which were not present in the controls, or whose total relative abundance in all newborn samples was more than fourfold as high as their total relative abundance in empty collecting swab negative controls. The sequences which failed to fulfill these requirements were removed from the data. For example, for the newborns:

```
### Remove potential contaminant sequences based on the .accnos file (list of sequence names) exported from Excel:
remove.seqs(accnos=nb.contaminants.accnos, fasta=calf.nb.fasta,
count=calf.nb.count_table)
```

All the cleaned subsets were then merged again for further processing and analysis. First, the negative controls were removed from all subsets except newborns, to prevent their multiplication:

```
### Remove negative controls from data subsets (for example, dam feces):
remove.groups(fasta=calf.df.pick.fasta, count=calf.df.pick.count_table, groups=NS1-NS2-NS3-NTC1-NTC2-NTC3)
```

Merging the count files was not readily possible in mothur, so it was performed in Excel using VLOOKUP for the various count tables and a joint, deduplicated list of sequence names. The fasta files were then also merged:

```
### Merge fasta files:
merge.files(input=calf.df.pick.pick.fasta-calf.dm.pick.pick.fasta-
calf.dv.pick.pick.fasta-calf.do.pick.pick.fasta-calf.wo.pick.pick.fasta-
calf.nb.pick.fasta, output=calf.all_cleaned.fasta)
```

Duplicated sequences were removed from the fasta file using awk.

OTU clustering and taxonomic classification

After decontamination, data processing was again continued according to mothur MiSeq SOP, to cluster the OTUs and generate the shared file. Rare OTUs (less than 250 sequences in the entire data) were then removed. Finally, taxonomic classification of OTUs was performed based on the RDP database.

Supplementary references

1 Finotello, F., Mastrorilli, E. & Di Camillo, B. Measuring the diversity of the human microbiota with targeted next-generation sequencing. *Brief Bioinform*, doi:10.1093/bib/bbw119 (2016).

2 Jost, L. Partitioning diversity into independent alpha and beta components. *Ecology* **88**, 2427-2439 (2007).

3 Chao, A., Chiu, C.-H. & Jost, L. Unifying species diversity, phylogenetic diversity, functional diversity, and related similarity and differentiation measures through Hill numbers. *Annu. Rev. Ecol. Evol. Syst.* **45**, 297-324, doi:10.1146/annurev-ecolsys-120213-091540 (2014).

4 Chao, A. *et al.* Rarefaction and extrapolation with Hill numbers: a framework for sampling and estimation in species diversity studies. *Ecol. Monogr.* **84**, 45-67, doi:10.1890/13-0133.1 (2014).

5 Chao, A. & Jost, L. Estimating diversity and entropy profiles via discovery rates of new species. *Methods Ecol. Evol.* **6**, 873-882, doi:10.1111/2041-210X.12349 (2015).

6 Hsieh, T. C., Ma, K. H. & Chao, A. iNEXT: an R package for rarefaction and extrapolation of species diversity (Hill numbers). *Methods Ecol. Evol.* **7**, 1451-1456, doi:10.1111/2041-210X.12613 (2016).

7 Jost, L. Entropy and diversity. *Oikos* **113**, 363-375, doi:10.1111/j.2006.0030-1299.14714.x (2006).

8 Tuomisto, H. A consistent terminology for quantifying species diversity? Yes, it does exist. *Oecologia* **164**, 853-860, doi:10.1007/s00442-010-1812-0 (2010).

9 Shanks, O. C. *et al.* Community structures of fecal bacteria in cattle from different animal feeding operations. *Appl. Environ. Microbiol.* **77**, 2992-3001, doi:10.1128/AEM.02988-10 (2011).

10 Tapio, I. *et al.* Oral samples as non-invasive proxies for assessing the composition of the rumen microbial community. *PLoS One* **11**, e0151220, doi:10.1371/journal.pone.0151220 (2016).

11 Dewhirst, F. E. *et al.* The human oral microbiome. *J. Bacteriol.* **192**, 5002-5017, doi:10.1128/jb.00542-10 (2010).

12 Swartz, J. D. *et al.* Characterization of the vaginal microbiota of ewes and cows reveals a unique microbiota with low levels of lactobacilli and near-neutral pH. *Front Vet Sci* **1**, 19, doi:10.3389/fvets.2014.00019 (2014).

13 Bicalho, M. L. *et al.* Dynamics of the microbiota found in the vaginas of dairy cows during the transition period: Associations with uterine diseases and reproductive outcome. *J. Dairy Sci.* **100**, 3043-3058, doi:10.3168/jds.2016-11623 (2017).

14 Nadkarni, M. A., Martin, F. E., Jacques, N. A. & Hunter, N. Determination of bacterial load by real-time PCR using a broad-range (universal) probe and primers set. *Microbiology (Reading, England)* **148**, 257-266 (2002).

15 Weisburg, W. G., Barns, S. M., Pelletier, D. A. & Lane, D. J. 16S ribosomal DNA amplification for phylogenetic study. *J. Bacteriol.* **173**, 697-703 (1991).
16 Pereira, P. A. B. *et al.* Oral and nasal microbiota in Parkinson's disease. *Parkinsonism Relat. Disord.* **38**, 61-67, doi:10.1016/j.parkreldis.2017.02.026 (2017).
17 Kearse, M. *et al.* Geneious Basic: an integrated and extendable desktop software platform for the organization and analysis of sequence data. *Bioinformatics* **28**, 1647-1649, doi:10.1093/bioinformatics/bts199 (2012).
18 Martin, M. Cutadapt removes adapter sequences from high-throughput sequencing reads. *EMBnet.journal; Vol 17, No 1: Next Generation Sequencing Data Analysis* (2011).
19 Brooks, J. P. *et al.* The truth about metagenomics: quantifying and counteracting bias in 16S rRNA studies. *BMC Microbiol.* **15**, 66, doi:10.1186/s12866-015-0351-6 (2015).
20 Edgar, R. C. & Flyvbjerg, H. Error filtering, pair assembly and error correction for next-generation sequencing reads. *Bioinformatics* **31**, 3476-3482, doi:10.1093/bioinformatics/btv401 (2015).
21 Kozich, J. J., Westcott, S. L., Baxter, N. T., Highlander, S. K. & Schloss, P. D. Development of a dual-index sequencing strategy and curation pipeline for analyzing amplicon sequence data on the MiSeq Illumina sequencing platform. *Appl. Environ. Microbiol.* **79**, 5112-5120, doi:10.1128/aem.01043-13 (2013).
22 Schloss, P. D. *MiSeq SOP*, <https://www.mothur.org/wiki/MiSeq_SOP> (2013).
23 Quast, C. *et al.* The SILVA ribosomal RNA gene database project: improved data processing and web-based tools. *Nucleic Acids Res.* **41**, D590-596, doi:10.1093/nar/gks1219 (2013).
24 Cole, J. R. *et al.* The Ribosomal Database Project: improved alignments and new tools for rRNA analysis. *Nucleic Acids Res.* **37**, D141-145, doi:10.1093/nar/gkn879 (2009).
25 Lukasik, P. *et al.* The structured diversity of specialized gut symbionts of the New World army ants. *Mol. Ecol.* **26**, 3808-3825, doi:10.1111/mec.14140 (2017).
